# H_2_S‐Releasing Versatile Montmorillonite Nanoformulation Trilogically Renovates the Gut Microenvironment for Inflammatory Bowel Disease Modulation

**DOI:** 10.1002/advs.202308092

**Published:** 2024-02-02

**Authors:** Ting Jin, Hongyang Lu, Qiang Zhou, Dongfan Chen, Youyun Zeng, Jiayi Shi, Yanmei Zhang, Xianwen Wang, Xinkun Shen, Xiaojun Cai

**Affiliations:** ^1^ School and Hospital of Stomatology Wenzhou Medical University Wenzhou 325027 China; ^2^ Department of Otolaryngology Ruian People's Hospital The Third Affiliated Hospital of Wenzhou Medical University Wenzhou 325016 China; ^3^ School of Biomedical Engineering Research and Engineering Center of Biomedical Materials Anhui Medical University Hefei 230032 China

**Keywords:** colitis, gut microbiota, hydrogen sulfide gas therapy, immunoregulation, peptide dendrimer nanogel

## Abstract

Abnormal activation of the intestinal mucosal immune system, resulting from damage to the intestinal mucosal barrier and extensive invasion by pathogens, contributes to the pathogenesis of inflammatory bowel disease (IBD). Current first‐line treatments for IBD have limited efficacy and significant side effects. An innovative H_2_S‐releasing montmorillonite nanoformulation (DPs@MMT) capable of remodeling intestinal mucosal immune homeostasis, repairing the mucosal barrier, and modulating gut microbiota is developed by electrostatically adsorbing diallyl trisulfide‐loaded peptide dendrimer nanogels (DATS@PDNs, abbreviated as DPs) onto the montmorillonite (MMT) surface. Upon rectal administration, DPs@MMT specifically binds to and covers the damaged mucosa, promoting the accumulation and subsequent internalization of DPs by activated immune cells in the IBD site. DPs release H_2_S intracellularly in response to glutathione, initiating multiple therapeutic effects. In vitro and in vivo studies have shown that DPs@MMT effectively alleviates colitis by eliminating reactive oxygen species (ROS), inhibiting inflammation, repairing the mucosal barrier, and eradicating pathogens. RNA sequencing revealed that DPs@MMT exerts significant immunoregulatory and mucosal barrier repair effects, by activating pathways such as Nrf2/HO‐1, PI3K‐AKT, and RAS/MAPK/AP‐1, and inhibiting the p38/ERK MAPK, p65 NF‐κB, and JAK‐STAT3 pathways, as well as glycolysis. 16S rRNA sequencing demonstrated that DPs@MMT remodels the gut microbiota by eliminating pathogens and increasing probiotics. This study develops a promising nanoformulation for IBD management.

## Graphical abstract

An H_2_S‐releasing montmorillonite nanoformulation (DPs@MMT) is developed by adsorbing diallyl trisulfide‐loaded peptide dendrimer nanogels onto the MMT surface. DPs@MMT can remodel intestinal mucosal immune homeostasis by activating Nrf2/HO‐1 and PI3K‐AKT pathways, inhibiting p38/ERK MAPK, p65 NF‐κB, and JAK‐STAT3 pathways along with HIF‐1‐induced glycolysis; repair mucosal barrier by activating RAS/MAPK/AP‐1 pathway; and modulate gut microbiota by eliminating pathogens and increasing probiotics.

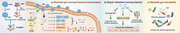

## 1 Introduction

Inflammatory bowel disease (IBD) is a group of chronic, non‐specific inflammatory disorders affecting the gastrointestinal tract, with unclear causes and characterized by recurring episodes. The most common forms of IBD are Crohn's Disease (CD) and Ulcerative Colitis (UC). Currently, IBD affects over 10 million people worldwide,^[^ ^1^, ^2^ ^]^ presenting significant treatment challenges, negatively impacting patients' quality of life, and placing a considerable financial burden on global healthcare systems.^[^ ^3^ ^]^ Traditional treatments like 5‐aminosalicylic acid, glucocorticoids, and azathioprine offer symptomatic relief but are limited by their non‐specific anti‐inflammatory effects and the potential for severe side effects.^[^ ^4^, ^5^ ^]^ In recent years, biologic agents such as tumor necrosis factor (TNF‐α) inhibitors, interleukin (IL) antagonists, and integrin receptor antagonists^[^ ^6^, ^7^ ^]^ have been introduced for IBD treatment. However, these therapies are often expensive, with limited response rates and significant side effects. For instance, a significant number of patients receiving infliximab show primary and secondary non‐responsiveness, and about a quarter of them experience serious complications, including acute inflammation, pneumonia, or cancer.^[^ ^8^ ^]^ Given these challenges, there is an urgent need to develop safer and more efficacious pharmaceuticals and therapeutic approaches for IBD management.

The pathogenesis of IBD is complex and not fully understood, but recent research highlights several key factors contributing to its development. These include sustained inflammatory responses,^[^ ^9^ ^]^ an excessive accumulation of reactive oxygen species (ROS), oxidative damage to the mucosal barrier,^[^ ^10^, ^11^, ^12^ ^]^ and gut microbiota dysbiosis,^[^ ^13^ ^]^ in its development. In IBD, the immune system within the colonic mucosa becomes abnormally active, characterized by the recruitment and infiltration of numerous immune cells. This activation leads to an intensified inflammatory response through the continuous secretion of chemokines, pro‐inflammatory cytokines, and ROS. The factors contributing to this heightened inflammatory state in IBD include: 1) Chemokines that recruit additional innate immune effector cells to the sites of inflammation;^[^ ^14^ ^]^ 2) Pro‐inflammatory cytokines that initiate and perpetuate inflammation;^[^ ^15^ ^]^ 3) High ROS levels that create a self‐sustaining cycle of inflammation and NF‐κB activation, further worsening inflammation and compromising mucosal permeability by inducing apoptosis in intestinal epithelial cells (IECs);^[^ ^16^ ^]^ 4) An imbalance in gut microbiota, particularly an increase in pathogenic bacteria, which can impair immune functions in the intestinal mucosa, disrupt epithelial permeability, or directly damage IECs.^[^ ^17^ ^]^ Given these insights, developing novel therapies that can simultaneously remodel intestinal mucosal immune homeostasis, repair the mucosal barrier, and regulate the gut microbiome: a concept termed the “trilogy of gut microenvironment renovation”, is crucial. Such therapies could significantly impact the effective management of IBD by addressing these key pathogenic mechanisms.

Gas therapy, a novel therapeutic approach, leverages gas molecules like hydrogen sulfide (H_2_S),^[^ ^18^, ^19^ ^]^ carbon monoxide (CO),^[^ ^20^, ^21^, ^22^ ^]^ nitric oxide (NO),^[^ ^23^, ^24^ ^]^ and hydrogen (H_2_)^[^ ^25^ ^]^ for disease treatment. These gases serve as crucial endogenous signaling molecules, providing therapeutic effects in various physiological and pathological contexts. H_2_S, in particular, has gained significant interest due to its remarkable antioxidant, anti‐inflammatory, and endothelial cell proliferation properties. Studies have shown that H_2_S can mitigate ROS‐induced apoptosis, deactivate MAPK signaling pathways, and reduce oxidative stress‐related damage in chronic conditions. For example, Wu et al. demonstrated H_2_S's efficacy in reducing tissue damage in chronic renal failure in rats.^[^ ^26^ ^]^ H_2_S also exhibits significant anti‐inflammatory effects, such as promoting M2 macrophage polarization and inhibiting the NF‐кB inflammatory signaling pathway.^[^ ^26^ ^]^ Additionally, research by Lin^[^ ^27^ ^]^ et al. and Chen^[^ ^15^ ^]^ et al. indicated that H_2_S can reduce leukocyte migration to inflammation sites and repair damage to the intestinal epithelial barrier, respectively. These findings suggest H_2_S's potential as an effective treatment for IBD, leveraging its antioxidant, anti‐inflammatory, and pro‐IEC proliferative effects. However, its clinical application faces challenges, particularly in controlled delivery due to its gaseous nature. To overcome this, various H_2_S donor molecules have been developed, though many lack precise controllability and sustained H_2_S release. NaHS and Na_2_S, for instance, are unstable and release H_2_S rapidly in physiological conditions,^[^ ^28^, ^29^ ^]^ raising cytotoxicity concerns. JK1 and JK2 provide acid‐sensitive H_2_S release but at an excessively fast rate.^[^ ^30^ ^]^ Diallyl trisulfide (DATS), an oil‐soluble organic sulfide from garlic, offers a more controlled and prolonged H_2_S release in the presence of glutathione (GSH). Theoretically, 1 mole of DATS can release 3 moles of H_2_S, enhancing its therapeutic efficiency.^[^ ^27^ ^]^ Moreover, DATS's inherent antimicrobial activity^[^ ^31^ ^]^ suggests its potential in regulating gut flora, making it a promising candidate for IBD treatment.

While DATS‐based H_2_S gas therapy holds promise for treating IBD through its potent antioxidant, anti‐inflammatory, and pro‐IECs proliferation effects, as well as its ability to modulate gut flora, the challenge lies in achieving controlled delivery of oil‐soluble DATS to targeted IBD sites. Peptide dendrimer nanogels (PDNs) are a cutting‐edge class of polymeric materials known for their excellent biocompatibility and capacity to carry both hydrophilic and hydrophobic drugs. Their adjustable surface chemistry allows for the attachment of various targeted ligands.^[^ ^20^, ^32^ ^]^ However, PDNs are not ideally suited for delivering gastrointestinal drugs due to their rapid degradation in acidic environments.^[^ ^33^ ^]^ In contrast, Montmorillonite (MMT), a naturally occurring phyllosilicate mineral, presents a more viable option. MMT features a nano‐layered structure with a negative charge,^[^ ^34^ ^]^ enabling it to bind specifically to and cover the positively charged, damaged mucosal surfaces.^[^ ^35^ ^]^ Its inability to cross the gastrointestinal barrier while maintaining normal colonic functions makes it an excellent choice for gastrointestinal drug delivery.^[^ ^36^ ^]^ MMT offers a large specific surface area and numerous negative charges, facilitating the electrostatic adsorption of positively charged nanoparticles.^[^ ^37^ ^]^ This property significantly enhances MMT's suitability as a carrier for gastrointestinal drugs, particularly in the context of IBD treatment.

**Scheme 1:**
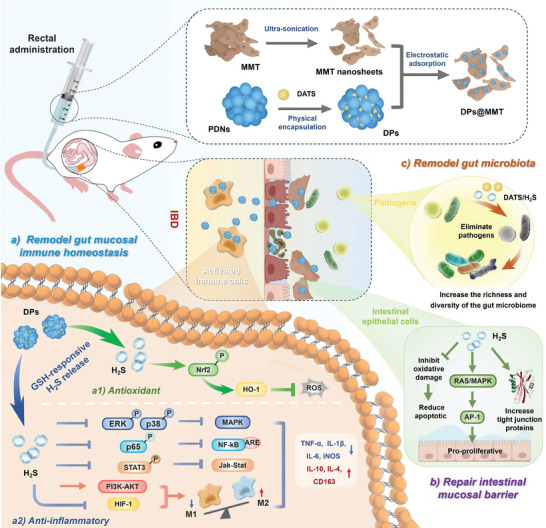
Illustration of DPs@MMT construction and its mechanisms in IBD treatment. a1) Antioxidant by activating the Nrf2/HO‐1 pathway, a2) Anti‐inflammatory by activating the PI3K/AKT pathway and inhibiting several key pathways including p38/ERK MAPK, p65 NF‐κB, JAK‐STAT3, and HIF‐1‐induced glycolysis, b) Mucosal barrier repair by protecting IECs from oxidative apoptosis, promoting IECs proliferation via the RAS/MAPK/AP‐1 pathway, and increasing TJPs expression; c) Modulate the gut microbiota by eliminating pathogens and increasing the richness and diversity of the gut microbiome.

Here, we have developed an advanced nanoformulation named DPs@MMT specifically tailored for the treatment of IBD. This formulation is capable of remodeling intestinal mucosal immune homeostasis, repairing the mucosal barrier, and modulating the gut microbiota. The preparation of DPs@MMT involves first encapsulating DATS within PDNs to form DATS‐loaded PDNs (abbreviated as DPs) and then electrostatically adsorbing these DPs onto the surface of MMT (**Scheme** **1**). Upon rectal administration, DPs@MMT can selectively bind to and cover the damaged intestinal mucosa, facilitating the accumulation of DPs at the site of IBD and subsequent internalization by activated immune cells. DPs release H_2_S intracellularly to initiate a range of therapeutic effects. These include potent antioxidant and anti‐inflammatory effects, and promotion of mucosal barrier repair. The DATS payload can also actively eradicate harmful bacteria prevalent at IBD sites. The in vivo efficacy of DPs@MMT has been rigorously tested in a mouse model of colitis. The results demonstrated significant therapeutic benefits and biocompatibility, underscoring the potential of DPs@MMT as an innovative and effective nanoformulation for IBD treatment.

## 2 Results and Discussion

### 2.1 Construction and Characterization of DPs@MMT

**Figure 1:**
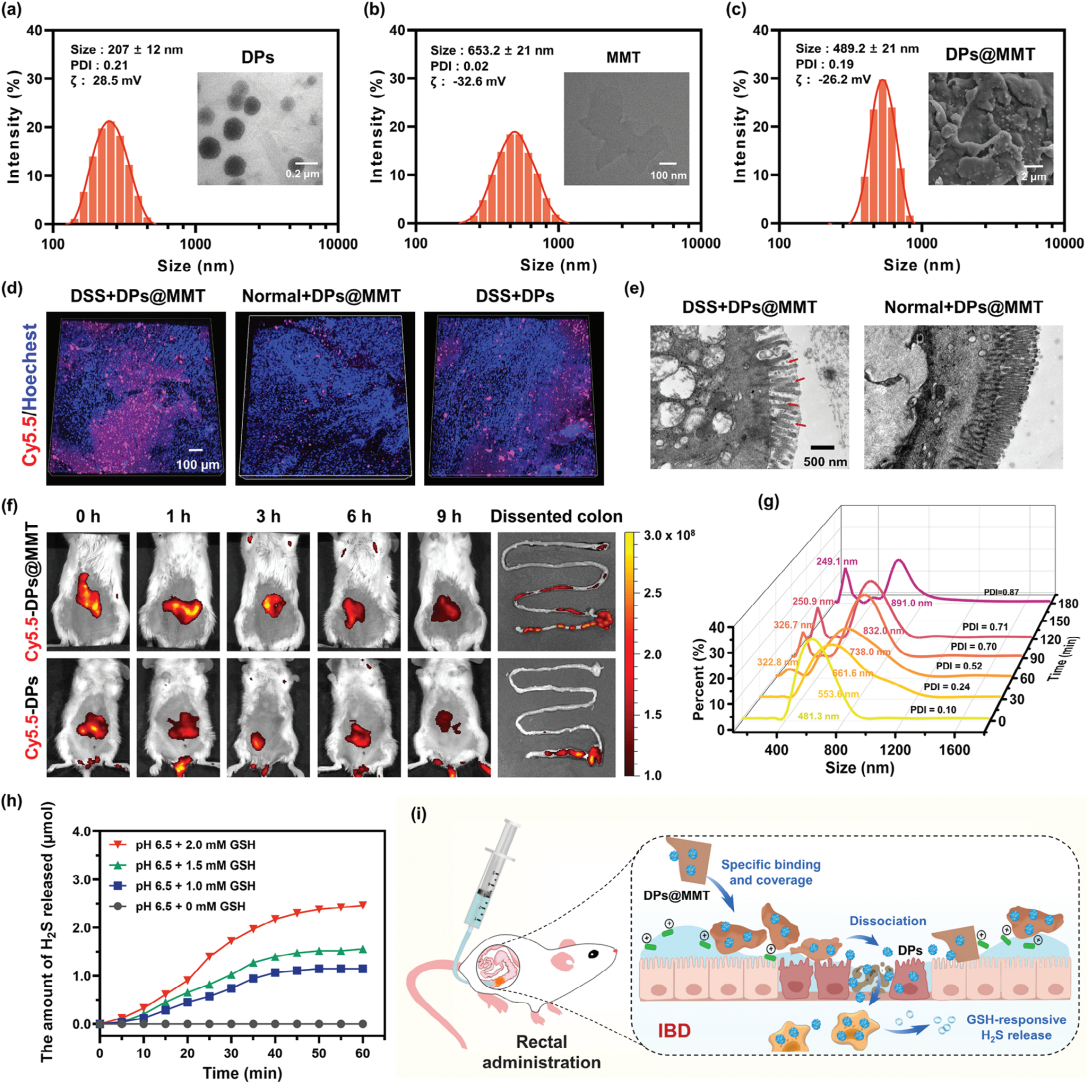
Physicochemical characterization of DPs@MMT: a–c) The particle size distributions, and TEM or SEM images of DPs, MMT, and DPs@MMT; d) CLSM images illustrate the *ex vivo* distribution of Cy5.5‐DPs@MMT and Cy5.5‐DPs in the colonic tissue of a mouse with colitis and a healthy mouse, observed 1 hour after rectal administration; e) Bio‐TEM images depict the gut mucosa of both inflamed and healthy mice following one hour of rectal administration of DPs@MMT or DPs; f) The biodistribution of Cy5.5‐DPs@MMT and Cy5.5‐DPs in the colon post rectal administration is presented, showingvarying time points; g) Variation of particle size and PDI of DPs@MMT during a 3‐h incubation in ACF; h) H_2_S release profiles of DPs@MMT in ACF with varying concentrations of GSH; i) Schematic illustration of specific binding and coverage; dissociation of DPs and GSH‐responsive release of H_2_S by DPs@MMT at the IBD site.

The fabrication of DPs@MMT was achieved through a complex multi‐stage process. Initially, an octa‐(3‐aminopropyl)silsesquioxane (OAS) core‐based generation three poly‐_l_‐lysine dendrimer (OAS‐G3‐Lys) underwent cross‐linking with 3,3′‐dithiodipropionate acid di(N‐hydroxysuccinimide ester) (DSP). This step resulted in the formation of OAS‐G3‐Lys‐based blank PDNs. Subsequently, DATS was physically encapsulated within these PDNs to produce the DPs. The final stage of the synthesis involved the electrostatic adsorption of these DPs onto the surface of montmorillonite (MMT), culminating in the creation of the DPs@MMT nanoformulation. The PDNs exhibited a hydrodynamic size of 240 ± 17 nm, a polydispersity index (PDI) of 0.19, and a positive ζ‐potential of ≈33.7 mV (Figure S1, Supporting Information). In order to maximize the encapsulation of DATS within the PDNs, we systematically tested three different DATS to PDNs feed ratios: 40%, 50%, and 60% (w/w). Both the loading content and efficiency of DATS progressively increased with higher feed ratios, reaching a maximum at 60% with a loading content of 30.1% and efficiency of 43.0% (Table S1, Supporting Information). Consequently, for all subsequent experimental procedures, DPs prepared at this optimal 60% feed ratio were utilized. These DPs exhibited a characteristic spherical morphology with an average diameter of 207 ± 12 nm (**Figure** **1**a), which was slightly smaller than the original PDNs, likely due to the tightening of the hydrophobic core caused by DATS encapsulation. For the effective adsorption of DPs onto MMT, a monolayer or few‐layer MMT with a substantially large specific surface area was prepared by an ultrasonic exfoliation technique. The resultant exfoliated MMT manifested a distinct layered structural morphology with a mean diameter of 653.2 ± 21 nm, a PDI of 0.02, and a negative ζ‐potential of −32.6 mV (Figure 1b). For the synthesis of the DPs@MMT nanoformulation, three distinct feed ratios of DPs to MMT (0.04/1, 0.05/1, and 0.1/1, w/w) were meticulously evaluated. The changes in particle size and ζ‐potential after adsorption of DPs on MMT indicated successful adsorption (Table S2, Supporting Information). The adsorption efficiency of DPs remained consistently high, ≈90%, across the varying feed ratios. This was accurately quantified using a standard curve derived from Cy5.5‐labeled DPs (Cy5.5‐DPs, Figure S2, Supporting Information). The adsorption content, however, decreased with increasing feed ratio, from about 9.2% at 0.04/1 to 3.8% at 0.1/1 (Table S2, Supporting Information). The reduction in MMT particle size post‐DPs adsorption is attributed to the positive charge of the DPs, which effectively lessens the aggregation of MMT flakes. Correspondingly, the ζ‐potential of the DPs@MMT complex showed an increase with the adsorption content of DPs. To maintain the optimal ζ‐potential and avoid it becoming positive, which could potentially weaken the adhesion of MMT to the positively charged damaged intestinal mucosa,^[^ ^38^ ^]^ a feed ratio of 0.05/1 was selected for DPs@MMT synthesis. Under these conditions, DPs@MMT achieved an average particle size of 489.2 ± 21 nm and a PDI of 0.19, with a retained negative ζ‐potential of −26.2 mV (Figure 1c). Scanning Electron Microscope (SEM) images further corroborated the successful preparation of DPs@MMT, displaying numerous spherical nanoparticles with an average size of ≈200 nm. Furthermore, DPs@MMT exhibited remarkable physiological stability, maintaining consistent particle size and PDI even after a seven‐day incubation in pH 7.4 PBS (Figure S3, Supporting Information).

Following the successful synthesis of DPs@MMT, its capability to selectively bind to and cover the positively charged damaged intestinal mucosa was evaluated using Confocal Laser Scanning Microscopy (CLSM). DPs@MMT demonstrated a pronounced ability to rapidly and specifically adhere to the damaged mucosal surface (Figure 1d). This was evidenced by the significant accumulation of Cy5.5‐DPs@MMT on the colonic tissue surface of mice afflicted with dextran sulfate sodium (DSS)‐induced colitis, particularly notable one hour after rectal administration. This accumulation was substantially greater than that observed on the normal colonic tissue of healthy mice. Additionally, the amount of Cy5.5‐DPs bound to the colonic tissue of mice with colitis was considerably less than that in the Cy5.5‐DPs@MMT‐treated group, reinforcing the inherent capacity of MMT to target and enshroud the positively charged damaged mucosa. Transmission Electron Microscopy (TEM) analysis of ultrathin colon tissue sections confirmed extensive binding and coverage of DPs@MMT on the damaged intestinal mucosa (highlighted by red arrows), contrasted by minimal adherence to healthy mucosal areas (Figure 1e). The retention dynamics of DPs@MMT at IBD sites in colitis mice were further investigated using in vivo imaging systems (IVIS). The results revealed sustained retention of DPs@MMT at the IBD site; vivid Cy5.5 fluorescence was still detectable in the colon of the Cy5.5‐DPs@MMT group 9 hours after administration, in stark contrast to the nearly undetectable fluorescence in the Cy5.5‐DPs group (Figure 1f). In sum, these observations indicate that the specific binding and coverage of MMT on the damaged intestinal mucosa markedly extends the residence time of DPs at IBD sites, a critical factor for the efficacious delivery of the DATS and H_2_S payloads.

Given the inability of MMT to penetrate the intestinal mucosal barrier^[^ ^36^ ^]^ and the predominant localization of activated immune cells within the intestinal lamina propria, it was crucial to determine whether DPs@MMT could dissociate at the damaged mucosal surface. This disassociation process is vital for allowing DPs to traverse the impaired mucosa, reach the lamina propria, and subsequently release H_2_S within the activated immune cells, thereby initiating antioxidant and anti‐inflammatory actions. To varify this, we investigated the dissociation behavior of DPs@MMT in artificial colonic fluid (ACF), primarily consisting of phosphate buffer with a pH of 6.5). The study aimed to monitor changes in particle size and PDI during the dissociation process. Upon 1 h of incubation in ACF, DPs@MMT began to dissociate slowly, indicated by the appearance of nanoparticles averaging 322.8 nm in diameter (Figure 1g). This observation suggested the gradual release of DPs from the DPs@MMT complex. With prolonged incubation, an increasing number of DPs dissociated from DPs@MMT, leading to the formation of larger aggregates of dissociated MMT nanoparticles, exceeding 800 nm in diameter. The rising PDI values corroborated the ongoing dissociation process. These findings suggest that after rectal administration, DPs@MMT first binds to and covers the damaged intestinal mucosa, subsequently dissociating to release DPs. Additionally, the study explored the release behavior of H_2_S from DPs and DPs@MMT, which is crucial for their efficacy in antioxidant and anti‐inflammatory activities, enhancement of IECs proliferation, and modulation of the gut microbiome. These are key factors for the effective treatment of IBD. The H_2_S release profile of DPs@MMT was assessed in ACF (pH = 6.5) supplemented with varying concentrations of glutathione (GSH) (0, 1, 1.5, and 2 mm). The GSH concentration at the IBD site, as measured in this study, was ≈1.55 mm, aligning with literature values.^[^ ^39^ ^]^ At 0 mm GSH, DPs@MMT exhibited minimal H_2_S release after one hour. However, at 1.0, 1.5, and 2.0 mm GSH, the cumulative H_2_S release reached 1.15, 1.56, and 2.46 µmol, respectively, after 1 h (Figure 1h). These results demonstrate that DATS within DPs@MMT displays a GSH‐responsive H_2_S release behavior, confirming its ability to effectively release H_2_S at the IBD site. Figure 1i presents a schematic representation of the specific binding and coverage of DPs@MMT at the IBD site, along with its gradual disassociation and GSH‐responsive H_2_S release mechanism.

### 2.2 Antioxidant and Anti‐Inflammatory Activities of DPs@MMT

**Figure 2:**
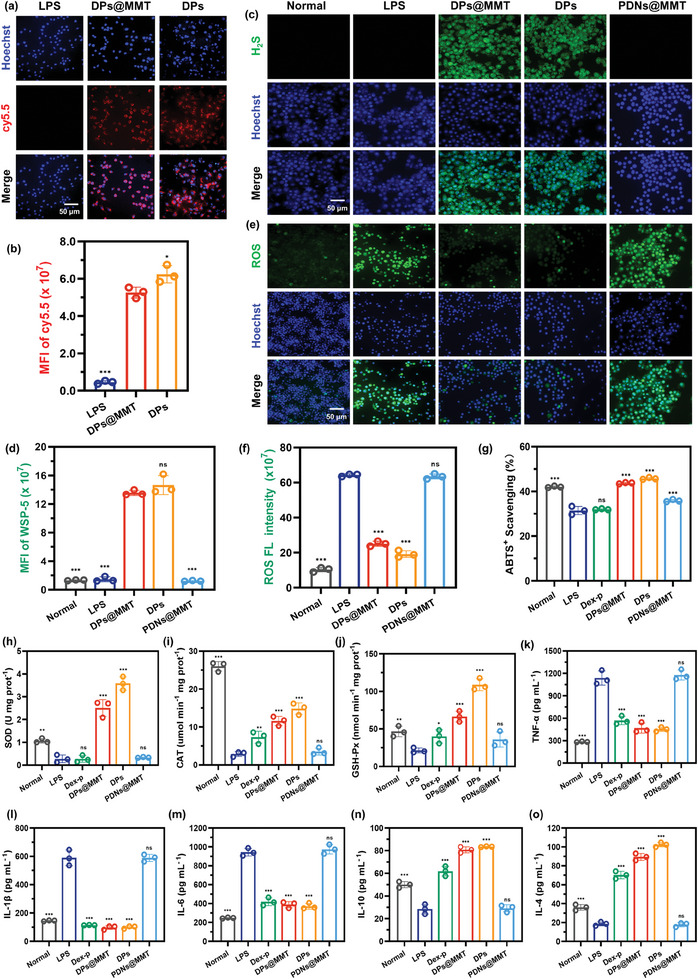
In vitro antioxidant and anti‐inflammatory activities of DPs@MMT. a,b) Cellular uptake of Cy5.5‐DPs@MMT and intracellular fluorescence intensity of Cy5.5; c,e) Intracellular imaging of H_2_S and ROS scavenging and d,f) Intracellular fluorescence intensity of WSP‐5 and DCF probes; g) The ABTS^+^ scavenging capacity and enzymatic activity levels of h) SOD, i) CAT, and j) GSH‐Px in DPs@MMT‐treated activated macrophages; k–o) The expression levels of TNF‐α, IL‐1β, IL‐6, IL‐10, and IL‐4 in DPs@MMT‐treated activated macrophages. In Figure b,d: DPs@MMT group versus other groups (*n* = 3); in Figures f‐o: LPS group versus other groups (*n* = 3), *p < 0.05, **p ≤ 0.01, ***p ≤ 0.001.

Before delving into its antioxidant and anti‐inflammatory capabilities, the biocompatibility of DPs@MMT was thoroughly evaluated. Both Raw264.7 and Caco‐2 cell lines, upon treatment with DPs@MMT and DPs, displayed minimal PI‐labeled dead cells, maintaining cell viability above 90%. This outcome, as evidenced in Figure S4 (Supporting Information), demonstrates the formulations' exceptional biocompatibility. A crucial aspect of DPs@MMT's functionality is its successful cellular uptake, a prerequisite for intracellular H_2_S release. Using CLSM, the cellular internalization of Cy5.5‐labeled DPs@MMT in lipopolysaccharides (LPS)‐activated RAW264.7 macrophages was observed. The cells treated with Cy5.5‐DPs@MMT (2 mg mL^−1^) exhibited intense red fluorescence (**Figure** **2**a), indicating efficient uptake. Notably, Cy5.5‐DPs (100 µg mL^−1^) demonstrated slightly higher cellular uptake efficiency compared to Cy5.5‐DPs@MMT. This difference, further substantiated by quantitative fluorescence intensity analysis of Cy5.5 (Figure 2b), could be attributed to the favorable charge and size characteristics of Cy5.5‐DPs for cellular entry. Following this effective cellular uptake, the intracellular H_2_S‐releasing capability of DPs@MMT was assessed. This assessment utilized Washington State Probe‐5 (WSP‐5), a dedicated fluorescent probe for H_2_S. Figure 2c,d show a marked green fluorescence in DPs@MMT‐treated macrophages, indicating a rapid and substantial release of H_2_S within the cells. This fluorescence is the result of WSP‐5 reacting swiftly with H_2_S. In comparison, DPs also displayed substantial intracellular H_2_S release, but significantly, activated macrophages treated with PDNs@MMT exhibited almost no H_2_S production. This striking difference underscores the unique H_2_S‐releasing property of DPs@MMT, setting it apart as an effective therapeutic agent.

The significant generation of H_2_S by DPs@MMT has been correlated with a marked decrease in intracellular ROS, as evidenced by the intense green fluorescence from dichlorofluorescein (DCF). This fluorescence, which arises when dichlorodihydrofluorescein diacetate (DCFH‐DA) is oxidized by ROS, was prominently seen in LPS‐induced activated macrophages. Conversely, in macrophages treated with DPs@MMT and DPs, this fluorescence was nearly absent (Figure 2e). The reduction of intracellular DCF fluorescence intensity by 75% and 81% for DPs@MMT and DPs respectively (Figure 2f) underscores their potent antioxidant capabilities. This antioxidant effect was further substantiated by the ABTS assay. The assay revealed that treatment with DPs@MMT and DPs significantly enhanced the total antioxidant capacity of the activated macrophages. Specifically, the scavenging capacity for ABTS^+^ radicals in cells treated with DPs@MMT and DPs was approximately two to three times higher compared to that in the PBS‐treated cells (Figure 2g). These results highlight the primary role of H_2_S in the antioxidant properties of these treatments, in contrast to PDNs@MMT, which neither scavenged ROS nor improved intracellular antioxidant capacity. Further studies delved into the mechanistic aspects of H_2_S's antioxidant action. It was found to upregulate the expression of key intracellular antioxidant enzymes, including superoxide dismutase (SOD), catalase (CAT), and glutathione peroxidase (GSH‐Px). Figures 2h–j show that the expression levels of these enzymes in the DPs@MMT group were significantly higher (10.1‐, 3.6‐, and 3.1‐fold respectively) than in the LPS group. This upregulation is crucial, given that activated macrophages typically produce high levels of superoxide anions and hydrogen peroxide (H_2_O_2_), which are central to the inflammatory response process.^[^ ^40^, ^41^ ^]^ The substantial increase in SOD, CAT, and GSH‐Px, known for their superoxide anion and H_2_O_2_ scavenging abilities, strongly indicates a robust reduction in intracellular ROS levels. Notably, the extraordinary ability of H_2_S to scavenge ROS may also be attributed to its direct reaction with single or double electron ROS.^[^ ^42^ ^]^ In contrast, other treatments like dexamethasone sodium phosphate (Dex‐p) and PDNs@MMT had a negligible effect on the expression of these crucial antioxidant enzymes, thus demonstrating limited ROS scavenging efficacy.

Upon verifying the exceptional antioxidant properties of DPs@MMT, its impact on the expression of inflammatory factors was evaluated using Enzyme‐Linked Immunosorbent Assay (ELISA). The study showed that activated macrophages significantly overexpressed key inflammatory factors, particularly TNF‐α, IL‐1β, and IL‐6. Levels of these cytokines were up to 1137, 589, and 944 pg mL^−1^, respectively (Figure 2k–m). These values were substantially elevated compared to those in normal macrophages, which recorded levels of 284, 145, and 247 pg mL^−1^, respectively. Dex‐p, a standard first‐line anti‐inflammatory dedication, effectively reduced the secretion of these inflammatory factors. Post Dex‐p treatment, the levels of TNF‐α, IL‐1β, and IL‐6 decreased to 570, 114, and 419 pg mL^−1^, respectively. Remarkably, DPs@MMT demonstrated even more pronounced therapeutic efficacy. It significantly lowered the expression levels of TNF‐α, IL‐1β, and IL‐6 to 469, 97, and 388 pg mL^−1^, respectively, closely aligning with the levels observed in normal macrophages. Furthermore, DPs@MMT's effects extended beyond the reduction of pro‐inflammatory cytokines. It notably increased the expression of anti‐inflammatory cytokines. In the activated macrophages treated with PBS, IL‐10 and IL‐4 levels were relatively low, at 28 and 18 pg mL^−1^, respectively (Figure 2n,o). In contrast, following treatment with DPs@MMT, these levels surged significantly to 80 and 89 pg mL^−1^. The Dex‐p group exhibited IL‐10 and IL‐4 levels of 61 and 70 pg mL^−1^, respectively, for comparison. Overall, these findings powerfully indicate that DPs@MMT achieves superior anti‐inflammatory effects by effectively suppressing the expression of pro‐inflammatory cytokines and simultaneously enhancing the expression of anti‐inflammatory cytokines. Its anti‐inflammatory capabilities were comparable to those of DPs but were more potent than those of Dex‐p. It is noteworthy that PDNs@MMT showed minimal anti‐inflammatory activity, confirming that the pronounced anti‐inflammatory effect of DPs@MMT is primarily attributed to the H_2_S released from the DATS component.

### 2.3 Elucidation of the Antioxidant and Anti‐Inflammatory Mechanisms of DPs@MMT

**Figure 3:**
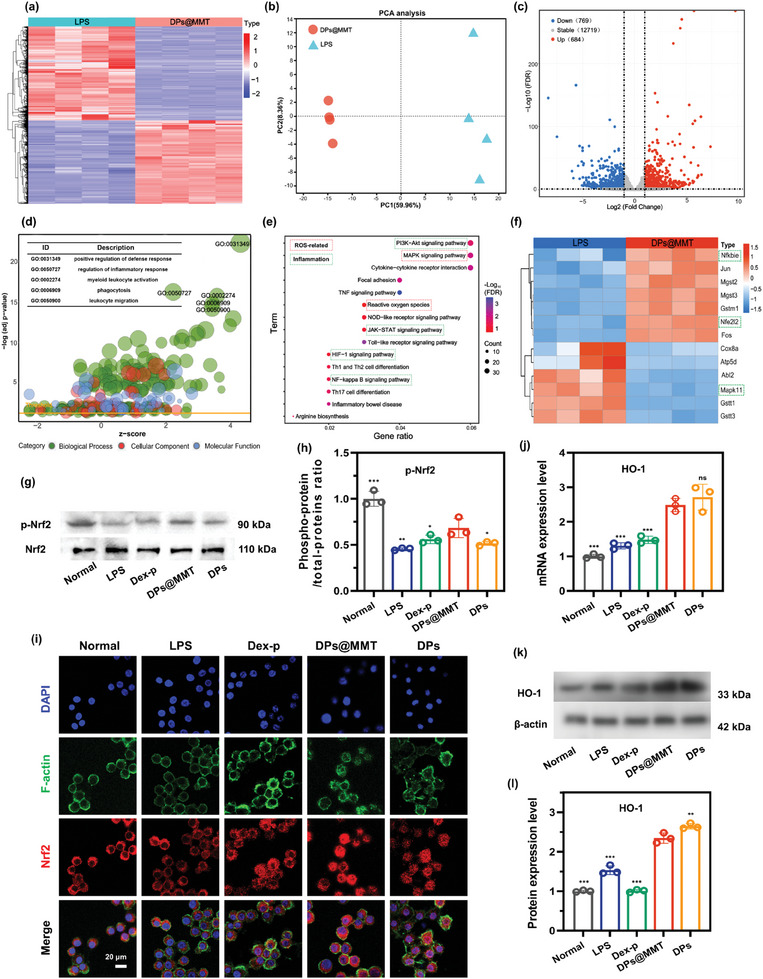
RNA‐seq analysis of activated macrophages treated with DPs@MMT. a) DEGs in LPS and LPS+DPs@MMT groups; b) PCA analysis of samples from LPS and DPs@MMT groups; c) Volcano plots for DEGs; d) GO and e) KEGG enrichment analysis of samples from LPS and DPs@MMT groups; f) Normalized heatmap showing the ROS‐related genes in LPS and DPs@MMT groups; g–h) Expression levels of p‐Nrf2 in DPs@MMT group; i) Intracellular location of Nrf2 in DPs@MMT group; j–l) mRNA and protein expression levels of HO‐1 in DPs@MMT group; In Figure h–k: DPs@MMT group versus other groups (*n* = 3), *p < 0.05, **p ≤ 0.01, ***p ≤ 0.001.

The comprehensive RNA sequencing (RNA‐seq) analysis on LPS‐activated macrophages treated with DPs@MMT or PBS revealed pivotal insights into the complex antioxidant and anti‐inflammatory mechanisms of DPs@MMT. The heatmap depicted in **Figure** **3**a vividly illustrates the stark contrast in gene expression profiles between the LPS and DPs@MMT groups, highlighting the significant impact of DPs@MMT on the macrophages' transcriptome. This difference is further underscored by the Principal Component Analysis (PCA), which shows distinct transcriptional variations induced by DPs@MMT treatment (Figure 3b). The volcano plot analysis, as shown in Figure 3c, is particularly revealing. It demonstrates that DPs@MMT treatment resulted in an upregulation of 684 genes and a downregulation of 769 genes compared to the LPS group, indicating significant alterations in gene expression. The Gene Ontology (GO) enrichment analysis, presented in Figure 3d, highlights that the differentially expressed genes (DEGs) are significantly enriched in biological processes related to the positive regulation of defense response (GO:00 31349) and the regulation of the inflammatory response (GO:00 50727). The positive Z‐scores in these categories suggest that DPs@MMT may play a favorable role in modulating immune responses. Moreover, the Kyoto Encyclopedia of Genes and Genomes (KEGG) enrichment analysis, shown in Figure 3e, further decodes the pathways through which DPs@MMT exerts its immunomodulatory effects. This analysis implies that the therapeutic actions of DPs@MMT are associated with its antioxidant activity, its capacity to inhibit the secretion of pro‐inflammatory cytokines, and its role in promoting the polarization of M1 macrophages toward the M2 phenotype.

The detailed RNA sequencing analysis elucidated in Figure 3f provides a comprehensive view of the specific gene alterations responsive to ROS following DPs@MMT treatment. Notably, there was a decrease in the expression of MAPK11 (p38) and an increase in NFKBIE (IκBε, an inhibitor of NF‐κB), indicating a substantial modulation of key signaling pathways. The downregulation of MAPK11 (p38) is critical because excessive ROS can trigger cell cycle arrest and apoptosis through the p38 MAPK pathway, as detailed in previous studies.^[^ ^43^, ^44^ ^]^ Concurrently, the upregulation of NFKBIE (IκBε) suggests a potential inhibition of NF‐κB activation. NF‐κB, a key player in inflammatory responses, can be restrained by IκBε, which forms a complex with NF‐κB and retains it in the cytoplasm,^[^ ^45^ ^]^ preventing its transcriptional activity. Thus, the alterations in the expression levels of these genes signify that DPs@MMT may effectively mitigate intracellular ROS production or scavenge existing ROS. Moreover, the significant increase in the expression of NFE2L2 (Nrf2) reinforces the notion that DPs@MMT enhances the body's antioxidant defense mechanisms. Nrf2 is renowned for its role in inducing the expression of various antioxidant enzymes and phase II detoxification enzymes,^[^ ^46^ ^]^ effectively combatting oxidative stress. The upregulation of Nrf2 indicates that DPs@MMT might facilitate Nrf2's nuclear translocation, thereby augmenting the cellular antioxidant response. Further validation of these findings was achieved through Western blot (WB) analysis and immunofluorescence (IF) staining assays, which demonstrated significant enhancement in the phosphorylation and nuclear translocation of Nrf2 in LPS‐induced activated macrophages treated with DPs@MMT (Figures 3g–i). Additionally, there was a marked upregulation in the expression of heme oxygenase‐1 (HO‐1) (Figures 3j–l), an enzyme critical for its antioxidant properties and therapeutic potential in IBD.^[^ ^47^ ^]^ In summary, these results suggest that DPs@MMT can effectively scavenge ROS, primarily by promoting the nuclear translocation of Nrf2 and increasing the expression of key antioxidant enzymes, including HO‐1. This mechanism underscores the potential of DPs@MMT as a powerful agent in combating oxidative stress and inflammation, particularly in the context of IBD treatment.

**Figure 4:**
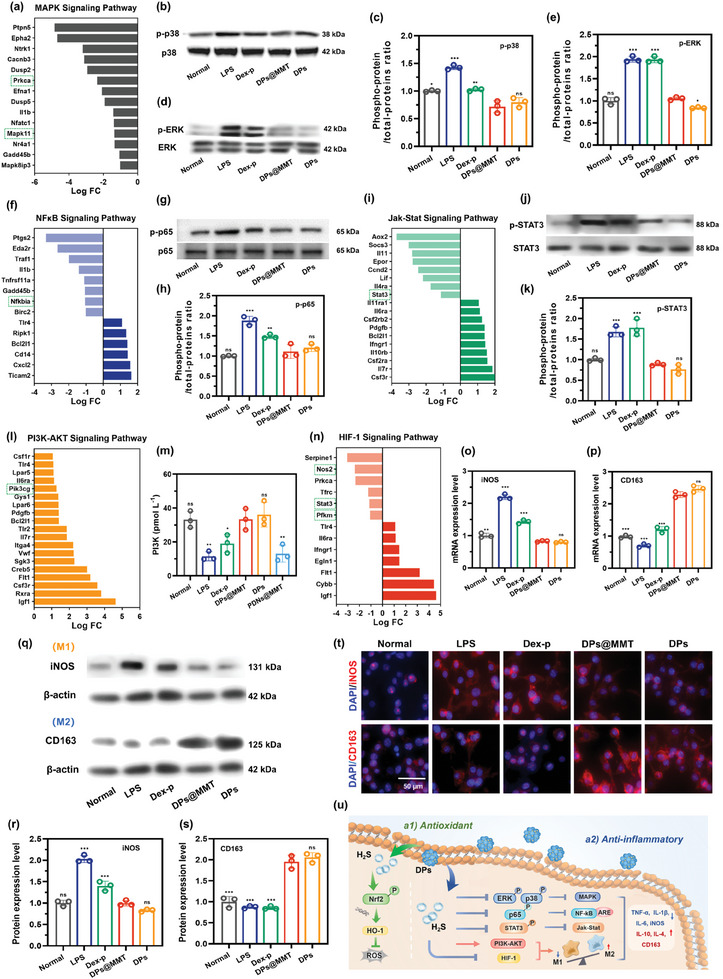
The anti‐inflammatory mechanisms of DPs@MMT. a) DEGs in the MAPK pathway and protein expression levels of b,c) p‐38 and d,e) p‐ERK in DPs@MMT group; f) DEGs in the NF‐κB pathway and protein expression levels of g‐h) p‐65 in DPs@MMT group; i) DEGs in the JAK‐STAT pathway and protein expression levels of j,k) STAT3 in DPs@MMT group; l) DEGs in the PI3K‐AKT pathway and m) PIK3 expression levels in DPs@MMT group; n) DEGs in the HIF‐1 pathway and o,p) mRNA and q–s) protein expression levels of iNOS and CD163 in DPs@MMT group; t) Immunofluorescence staining of iNOS‐positive and CD163‐positive cells in the DPs@MMT group; u) Schematic of the antioxidant and anti‐inflammatory effects of DPs@MMT. All statistical analysis were compared between DPs@MMT group and other groups (*n* = 3), *p < 0.05, **p ≤ 0.01, ***p ≤ 0.001.

Upon elucidating the potent antioxidant properties of DPs@MMT, we extended our investigation to its anti‐inflammatory mechanisms. KEGG enrichment analysis linked DPs@MMT's anti‐inflammatory activity to several signaling pathways, notably MAPK (**Figure** **4**a), NF‐κB (Figure 4f), JAK‐STAT (Figure 4i), PI3K‐AKT (Figure 4l), and HIF‐1 (Figure 4n). The MAPK pathway, recognized as a classic pro‐inflammatory cascade, is closely associated with the phosphorylation events of p38, ERK, and JNK. Notably, PRKCA (protein kinase C‐alpha, PKCα), which modulates both MAPK and NF‐κB pathways, is known to induce phosphorylation of p38 and ERK, as well as activate IκBε. Treatment with DPs@MMT significantly diminished the expression levels of p38 and PKCα (Figure 4a), indicating an effective suppression of the MAPK pathway. This was further corroborated by WB analysis, which demonstrated the inhibition of p38 and ERK phosphorylation by DPs@MMT, showing comparable to efficacy to DPs and superior effectiveness to Dex‐p (Figure 4b–e). The NF‐κB pathway, another pivotal inflammatory pathway, involves several genes including NFKBIA (IκBε). The phosphorylation and subsequent degradation of IκBε lead to the activation of p65/p50, triggering the production of pro‐inflammatory cytokines.^[^ ^48^, ^49^ ^]^ A notable decrease in IκBε expression (Figure 4f) by DPs@MMT underscores its substantial inhibition of the NF‐κB pathway. WB analysis supported these findings, displaying a marked inhibition of p65 phosphorylation by DPs@MMT, with an inhibitory effect comparable to that of DPs but stronger than that of Dex‐p (Figure 4g,h). The JAK‐STAT pathway plays a key role in both adaptive and innate immune responses, particularly in inflammatory bowel disease (IBD).^[^ ^50^ ^]^ Excessive activation of STAT3 is linked to the progression of IBD and colitis‐associated colorectal cancer.^[^ ^51^, ^52^ ^]^ The reduction in STAT3 expression (Figure 4i) following DPs@MMT treatment points to its potential in curbing the JAK‐STAT pathway. WB analysis validated this observation, showing significant inhibition of STAT3 phosphorylation by DPs@MMT, which was more efficacious compared to Dex‐p (Figure 4j,k).

In addition to its inhibitory effects on the MAPK, NF‐κB, and JAK‐STAT3 pathways, DPs@MMT treatment significantly activates the PI3K‐AKT pathway. This activation is evidenced by the upregulated expression of PIK3CG (PI3Kγ), a crucial regulator in immune cell function and a therapeutic target for inflammatory diseases.^[^ ^53^ ^]^ ELISA results (Figure 4m) confirm this, showing a marked increase in PI3K expression post DPs@MMT treatment. Importantly, PI3K‐AKT pathway activation encourages macrophage polarization toward the anti‐inflammatory M2 phenotype. This shift is demonstrated by the significant reduction in STAT3 and NOS2 expression levels (Figure 4n), suggesting a move away from the glycolysis‐dependent M1 macrophage metabolism. The downregulation of STAT3, in particular, hinders the activation of the HIF‐1 signaling pathway,^[^ ^54^ ^]^ thus suppresing glycolytic activity within these cells. Moreover, a marked decrease in PFKM expression (Figure 4n), a key glycolysis enzyme, indicates a reduction in glycolytic processes in DPs@MMT‐treated macrophages, leading to fewer M1 macrophages. Furthermore, the substantial decrease in NOS2 (iNOS) expression, a definitive M1 macrophage marker, underscores a significant reduction in the M1 macrophage population in DPs@MMT‐treated samples. Supporting this, results from Figures S5 and S6 (Supporting Information), and Figure 4o–t compellingly demonstrate that DPs@MMT treatment effectively induces macrophage polarization toward the M2 phenotype. This is supported by several key observations: 1) In the DPs@MMT group, cells showed remarkable elongation, with maximum elongation reaching 3.47, compared to 1.23 in the LPS group. The DPs@MMT group also had fewer synapses, averaging 2.67, significantly lower than the LPS group's average of 5.34 (Figure S5a–c, Supporting Information). 2) There was a marked decrease in iNOS expression and an increase in CD163 expression in the DPs@MMT group (Figures 4o–s), indicating a shift toward the M2 phenotype. 3) The DPs@MMT group displayed a significant reduction in red fluorescence of iNOS and an increase in red fluorescence of CD163 (Figure 4t), further confirming the phenotypic shift. 4) A notable 20.18% decrease in macrophages labeled with iNOS and a 26.95% increase in macrophages labeled with CD206 antibodies were observed in the DPs@MMT group compared to the LPS group (Figure S6, Supporting Information).

Together, these findings, as detailed in Figures 3 and 4, elucidate the mechanisms behind the antioxidant and anti‐inflammatory actions of DPs@MMT. The formulation not only enhances antioxidant activity through the nuclear translocation of Nrf2 and induction of HO‐1 expression but also exhibits strong anti‐inflammatory effects by inhibiting the p38/ERK MAPK, p65 NF‐κB, and JAK‐STAT3 pathways. Furthermore, it encourages macrophage polarization toward the M2 phenotype by activating the PI3K‐AKT pathway and suppressing glycolysis in M1 macrophages, as illustrated in Figure 4u.

### 2.4 Intestinal Mucosal Barrier Repair and Pathogen Elimination with DPs@MMT

**Figure 5:**
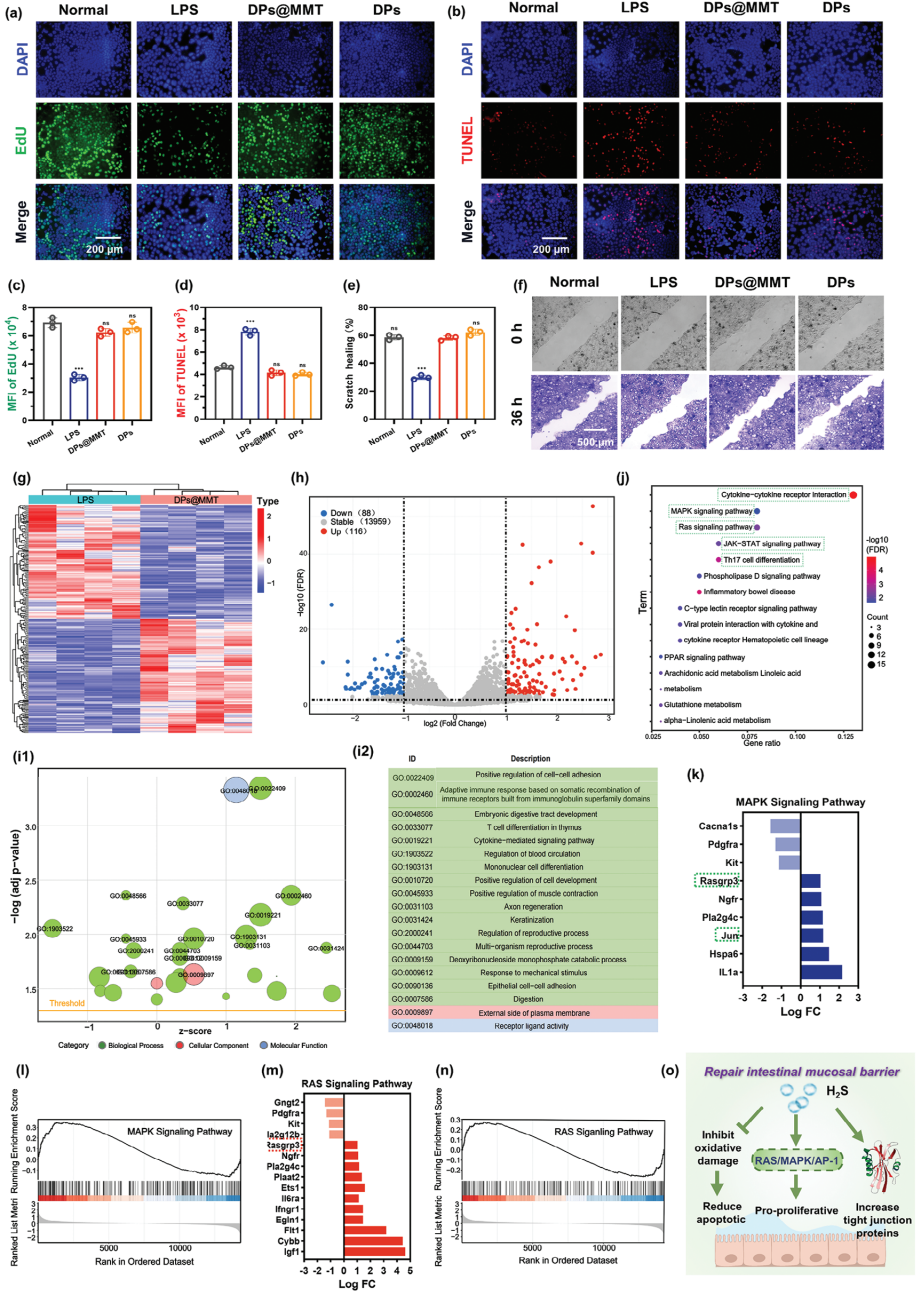
Effect of DPs@MMT on intestinal mucosal barrier repair and its underlying mechanisms. a,c) EdU staining, b,d) TUNEL staining, and e,f) Scratch healing of Caco2 cells post various treatments; g) DEGs in the LPS and LPS+DPs@MMT groups; h) Volcano plots for DEGs between LPS and LPS+DPs@MMT groups; i) GO and j) KEGG enrichment analysis of samples derived from LPS and LPS+DPs@MMT groups; k) DEGs in the MAPK pathway; l) GSEA of response to the MAPK pathway between LPS and LPS+DPs@MMT groups; m) DEGs in the RAS pathway; n) GSEA of response to the RAS pathway between LPS and LPS+DPs@MMT groups; o) Schematic illustration of the intestinal mucosal barrier repair effects of DPs@MMT. In Figure c,d: normal group versus other groups (*n* = 3); in Figure e: DPs@MMT group versus other groups (*n* = 3), ***p ≤ 0.001.

Elevated levels of ROS and inflammatory mediators are primary contributors to damage in the intestinal mucosal barrier.^[^ ^55^ ^]^ Given the remarkable antioxidant and anti‐inflammatory capabilities of DPs@MMT, we explored its potential in repairing this crucial barrier. Utilizing Caco‐2 cells, a widely recognized model for the human colorectal epithelium due to their differentiation and fusion properties, we evaluated the anti‐apoptotic, pro‐proliferative, and wound‐healing effects of DPs@MMT. The study revealed that Caco‐2 cells exposed to culture media from LPS‐induced activated macrophages treated with PBS exhibited significant apoptosis and reduced proliferation, evidenced by a high prevalence of TUNEL‐positive cells and a low count of EdU‐positive cells (**Figure** **5**a–d). In stark contrast, DPs@MMT treatment led to a marked decrease in apoptosis and a substantial increase in cell proliferation, underscoring its protective role against cellular damage induced by ROS and inflammation. Furthermore, DPs@MMT treatment notably accelerated wound healing in Caco‐2 cell cultures, as indicated by a significantly smaller residual scratch area compared to the control group (Figure 5e,f). These findings suggest that DPs@MMT not only effectively shields Caco‐2 cells from apoptosis in environments rich in ROS and inflammatory factors but also plays a crucial role in maintaining the integrity of the intestinal mucosal barrier. This barrier integrity is vital in preventing further exacerbation in conditions like IBD.

While H_2_S is recognized for its antioxidant and anti‐inflammatory properties, its impact on cell proliferation, especially in Caco‐2 cells, remains less explored. This study, therefore, delved into the proliferative mechanism of DPs@MMT on Caco‐2 cells through RNA‐seq analysis. The heatmap in Figure 5g shows significant transcriptomic differences between the LPS and DPs@MMT groups, including the upregulation of 116 genes and downregulation of 88 genes post DPs@MMT treatment (Figure 5h). GO analysis linked the DEGs to immune response (GO:0002460), cytokine‐mediated signaling (GO:0019221), and cell development (GO:0010720) (Figure 5i), all indicating activated proliferation pathways in Caco‐2 cells upon DPs@MMT treatment. Furthermore, KEGG enrichment analysis (Figure 5j) identified the RAS, MAPK, Th17 cell differentiation, JAK‐STAT pathways, and cytokine‐cytokine receptor interactions as key to DPs@MMT's proliferative effect. Gene Set Enrichment Analysis (GSEA) highlighted the pivotal role of the MAPK pathway in this effect (Figure 5k,l). The MAPK pathway, crucial for cell proliferation,^[^ ^56^ ^]^ is activated by RAS,^[^ ^57^ ^]^ influenced by RASGPR3. RASGPR3 physiologically activates RAS family members in response to growth factors or cytokines.^[^ ^58^, ^59^ ^]^ AP‐1, a key transcription factor in the RAS/MAPK pathway known for its anti‐apoptotic and pro‐proliferative actions,^[^ ^60^ ^]^ is enhanced by H_2_S,^[^ ^61^, ^62^ ^]^ as evidenced by the upregulation of RASGRP3 and JUN (a subunit of AP‐1) (Figure 5k–n). This suggests that DPs@MMT exerts its effects via the RAS/MAPK pathway and AP‐1 enhancement. Additionally, the cell‐cell adhesion pathway (GO:0022409 and GO:0090136) was notably regulated post DPs@MMT treatment (Figure 5i1,i2), including the upregulation of EST1 (Figure S7, Supporting Information), linked to TJPs expression.^[^ ^63^ ^]^ Intriguingly, H_2_S has been shown to inhibit MMP‐9 activity, involved in TJPs degradation, by restraining p65 NF‐κB activation.^[^ ^64^ ^]^ This is consistent with our findings in in Figure 4f–h, which confirm H_2_S's role in inhibiting p65 NF‐κB. Collectively, Figures 3, 4, 5 suggest that DPs@MMT's reparative effects on the intestinal mucosal barrier are due to its antioxidant and anti‐inflammatory properties, protecting Caco‐2 cells from oxidative apoptosis, and promoting cell proliferation via the RAS/MAPK/AP‐1 pathway and enhancing TJPs expression (Figure 5o).

The role of abnormal flora in ulcerative colitis is critical, particularly due to the invasion of the intestinal mucosa by pathogenic bacteria and endotoxins, which increase mucosal permeability.^[^ ^65^ ^]^ Our research goes beyond confirming DPs@MMT's protective role against apoptosis in IECs triggered by ROS and inflammatory agents and its promotion of rapid IEC proliferation. We explored the effectiveness of DPs@MMT in eliminating pathogenic bacteria prevalent at IBD sites. DATS, known for its ability to disrupt bacterial cell membranes and impede bacterial membrane transport systems, is recognized for its potent antimicrobial properties.^[^ ^31^, ^66^ ^]^ We assessed DPs@MMT's antimicrobial activity against prevalent IBD pathogens: *Staphylococcus aureus (S. aureus), Citrobacter rodentium (C. rodentium), and Escherichia coli (E. coli)*, using colony counting and live/dead bacterial staining assays. The results, as depicted in Figure S8 (Supporting Information), revealed that an 8‐h incubation with DPs@MMT led to Propidium Iodide (PI) labeling of nearly all bacterial cells of *S. aureus, C. rodentium, and E. coli*, characterized by intense red fluorescence and negligible green fluorescence. Colony counting data showed significant reductions in survival rates for these pathogens in the DPs@MMT group: 0.01% for *S. aureus*, 0.07% for *C. rodentium*, and 0.34% for *E. coli* (Figure S9, Supporting Information). These results highlight DPs@MMT's substantial antimicrobial capability, suggesting its potential to effectively combat a variety of pathogenic bacteria at IBD sites. Notably, both colony counting and live/dead staining assays indicated minimal antimicrobial activity in PDNs@MMT, hinting that the antimicrobial strength of DPs@MMT is primarily attributed to DATS.

### 2.5 Treatment Effect of DPs@MMT in DSS‐Induced Colitis Mouse Model

**Figure 6:**
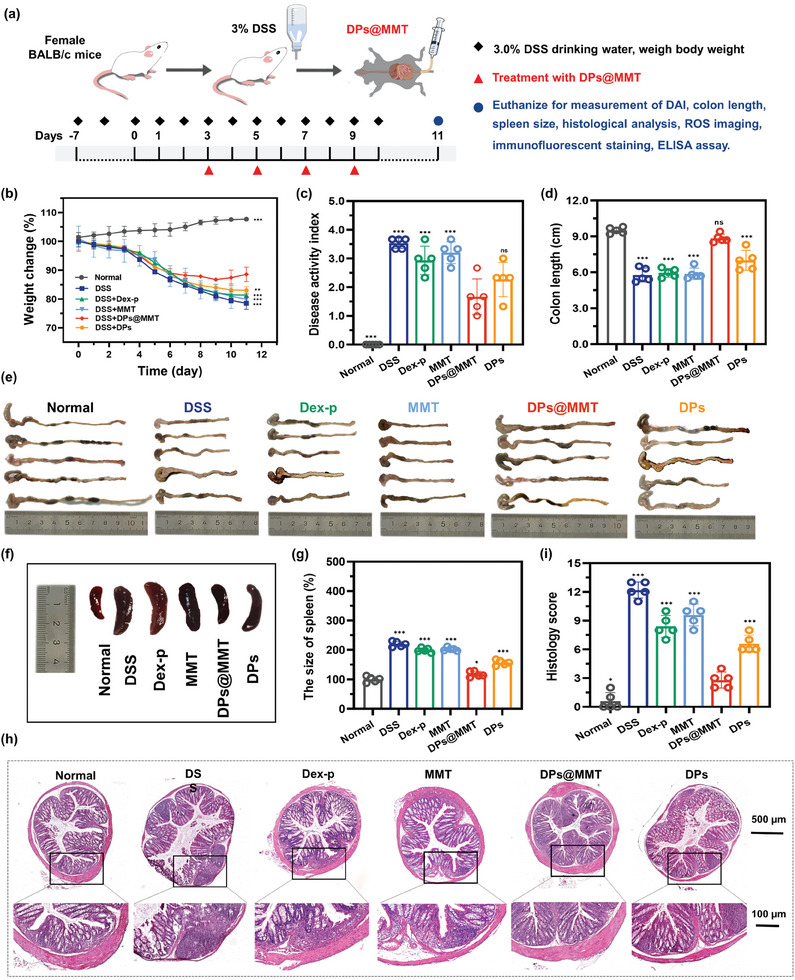
Therapeutic effect of DPs@MMT in mice with DSS‐induced colitis. a) Experimental protocol; b) Daily changes in body weight; c) DAI index, and d,e) Colon lengths, and f,g) Spleen size of mice at the end of treatment post various treatments; h) H&E staining images of colon tissues; i) Hisotology score on day 11. In Figure b,c and i: DPs@MMT group versus other groups (*n* = 5); in Figure d,g: normal group versus other groups (*n* = 5), **p ≤ 0.01, ***p ≤ 0.001.

Driven by the promising in vitro results of DPs@MMT in terms of its antioxidant, anti‐inflammatory, and intestinal mucosal barrier repair capabilities, along with its prolonged adhesion to damaged mucosal surfaces in IBD sites, we progressed to assess its therapeutic potential in an in vivo setting. This was conducted using a DSS‐induced colitis mouse model. To induce colitis, mice were given water containing 3% DSS, with close observation for the onset of bloody stools, typically around the seventh day. The treatment protocol continued with 3% DSS water consumption until the end of the study on day 11. We established several control groups for this study: healthy mice (normal group), mice with colitis treated with PBS (DSS group), mice with colitis treated with DPs (DPs group), along with a positive control group using Dex‐p, a commonly used IBD treatment known for its high relapse rate and wide range of side effects. MMT, often used in clinical settings to treat diarrhea, was included as another control. The treatment regimen for the Dex‐p, MMT, DPs, and DPs@MMT groups involved administering a single rectal dose of 150 µL of Dex‐p (75 µg mL^−1^), MMT (30 mg mL^−1^), DPs (1.5 mg mL^−1^), or DPs@MMT (30 mg mL^−1^) respectively, on days 3, 5, 7, and 9 (**Figure** **6**a). The therapeutic response was evaluated through body weight changes, colon length and spleen size measurements, Disease Activity Index (DAI) assessment, and histologic scoring.

Throughout the treatment period, a gradual decline in body weight was observed nearly all colitis‐afflicted mice (Figure 6b). The DSS group experienced a notable 20% weight reduction. Meanwhile, the Dex‐p, MMT, and DPs groups showed slightly less weight loss, at 19%, 19%, and 17% respectively by day 11. Impressively, the DPs@MMT group demonstrated the least weight loss, with a gradual recovery beginning after day nine and only a 12% loss by day 11 (Figure 6b). In DAI assessments, the DPs@MMT group achieved a significantly lower score of 1.6, compared to the higher scores in the DSS (3.5), Dex‐p (2.9), MMT (3.2), and DPs (2.3) groups (Figure 6c). An *ex vivo* analysis showed that the DSS, Dex‐p, MMT, and DPs groups experienced average colon length reduction of 39.2%, 37.3%, 38.1%, and 26.3%, respectively, in contrast to a minimal reduction of 6.9% in the DPs@MMT group (Figure 6d,e). Spleen size analysis indicated no significant enlargement in the DPs@MMT group, similar to the healthy group. Conversely, the DSS, Dex‐p, MMT, and DPs groups showed considerable spleen enlargement, being 2.2‐, 2.0‐, 2.0‐, and 1.6‐times larger, respectively, than the healthy group (Figure 6f,g). Hematoxylin and eosin (H&E) staining for colonic damage assessment revealed effective preservation of colonic epithelial integrity and minimal inflammatory cell infiltration in the DPs@MMT‐treated mice, resulting in a low damage score of 2.8. In contrast, the DSS, Dex‐p, MMT, and DPs groups exhibited significantly higher scores (12.2, 8.4, 9.6, and 6.6, respectively), with severe crypt destruction and extensive immune cell infiltration (Figure 6h,i). These findings highlight the potent protective effects of DPs@MMT against DSS‐induced colitis. Additionally, DPs@MMT's excellent biocompatibility was demonstrated by the minimal occurrence of occult blood in treated mice (Figure S10, Supporting Information), along with stable red blood cell count, white blood cell count, and hemoglobin levels: crucial markers associated with H_2_S poisoning (Figure S11, Supporting Information).^[^ ^67^ ^]^ No significant damage to vital organs was observed (Figure S12, Supporting Information). In summary, these results suggest DPs@MMT as an exceptionally promising nanoformulation for IBD management, outperforming Dex‐p across all metrics and highlighting its potential for clinical application. While DPs showed some therapeutic efficacy, surpassing Dex‐p, it did not reach the performance level of DPs@MMT, emphasizing MMT's role in enhancing the therapeutic impact of DPs@MMT in IBD treatment.

**Figure 7:**
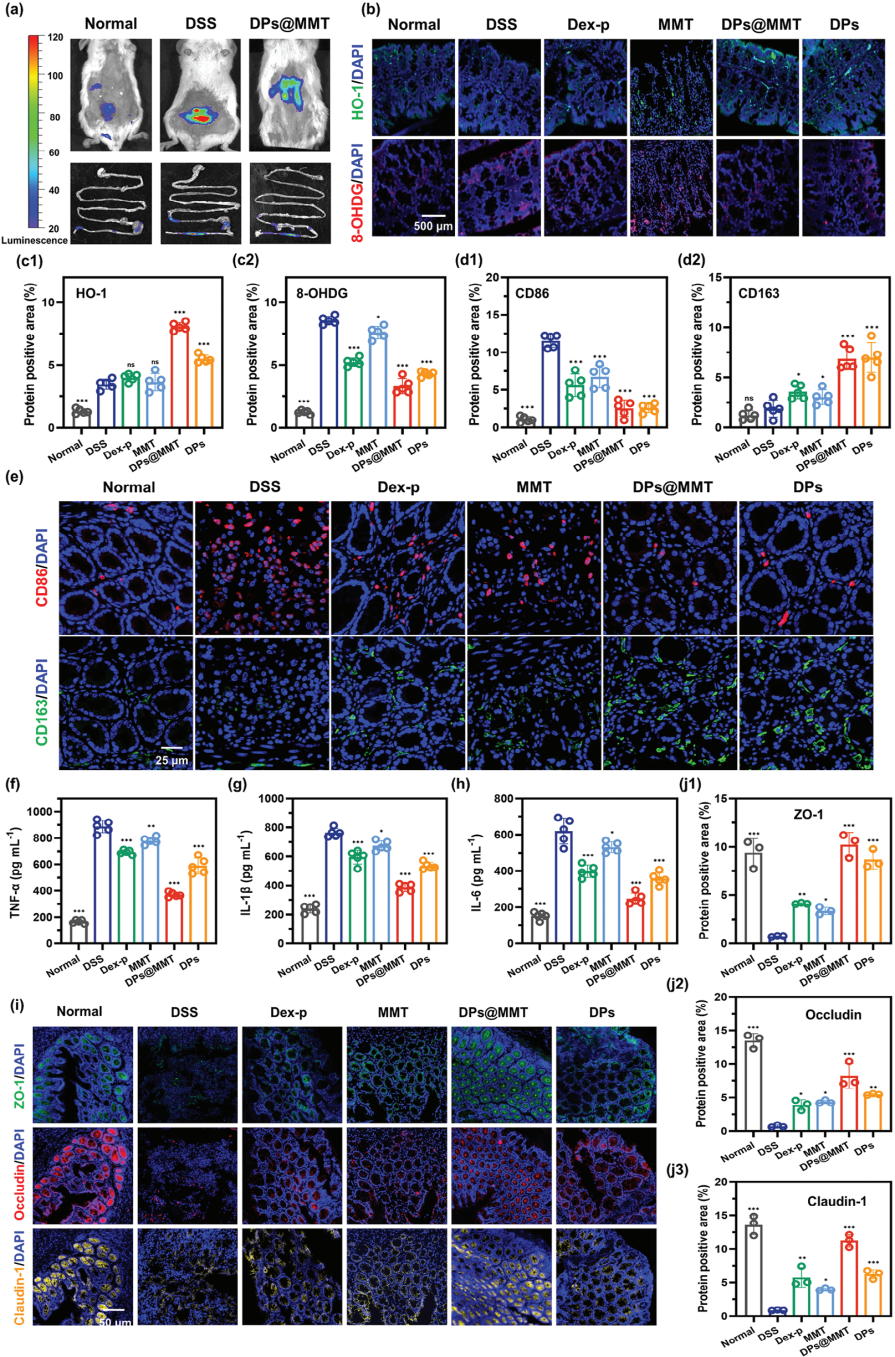
In vivo antioxidant, anti‐inflammatory, and mucosal barrier repair effects of DPs@MMT. a) ROS scavenging; b,c) Expression levels of HO‐1 and 8‐OHDG in colonic tissue; d,e) CD86‐positive and CD163‐positive cells in colonic tissue; f–h) Inflammatory cytokine expression levels in colonic tissue; i,j) Expression levels of TJPs in colonic tissue; All statistical analyses compare between DSS group and other groups (*n* = 5), *p < 0.05, **p ≤ 0.01, ***p ≤ 0.001.

Our in vitro studies have already demonstrated the outstanding antioxidant, anti‐inflammatory, and intestinal mucosal barrier repair properties of DPs@MMT. To determine the significance of these properties in its therapeutic efficacy for IBD, we investigated DPs@MMT's in vivo ability to scavenge ROS, mitigate oxidative stress, suppress inflammatory cytokine secretion, and promote M2 macrophage polarization at IBD sites. Using the L‐012 chemiluminescent probe, which fluoresces only in the presence of high levels of ROS at inflammatory sites,^[^ ^18^ ^]^ we observed marked ROS scavenging by DPs@MMT at IBD sites. **Figure** **7**a shows intense fluorescence in the DSS group, indicative of high ROS levels, which notably diminishes post‐treatment with DPs@MMT, implying significant ROS reduction at the IBD site. Additionally, DPs@MMT alleviated oxidative stress‐induced DNA damage, as indicated by the upregulation of HO‐1 protein expression and downregulation of 8‐OHDG, a marker of oxidative DNA damage, in treated colonic tissues (Figure 7b,c). The in vivo anti‐inflammatory effects of DPs@MMT were confirmed through immunofluorescence staining of colon tissues and cytokine quantification. Figure 7d,e illustrate the inhibition of CD86‐positive M1 macrophage differentiation and the enhancement of CD163‐positive M2 macrophage differentiation post‐treatment. Furthermore, the significantly lower levels of TNF‐α, IL‐1β, and IL‐6 in the DPs@MMT group compared to the DSS group point to effective inflammation suppression (Figures 7f‐h). DPs@MMT treatment also notably increased the expression of TJPs such as ZO‐1, occludin, and claudin‐1, essential for intestinal barrier integrity. The fluorescence intensities of these TJPs were considerably higher in the DPs@MMT group than in other groups (Figure 7i,j). In conclusion, these findings confirm that DPs@MMT exerts strong antioxidant, anti‐inflammatory, and mucosal barrier repair effects in vivo. These attributes are crucial for its ability to regulate immune homeostasis in the intestinal mucosa, underscoring its potential as an effective therapeutic agent for IBD management.

**Figure 8:**
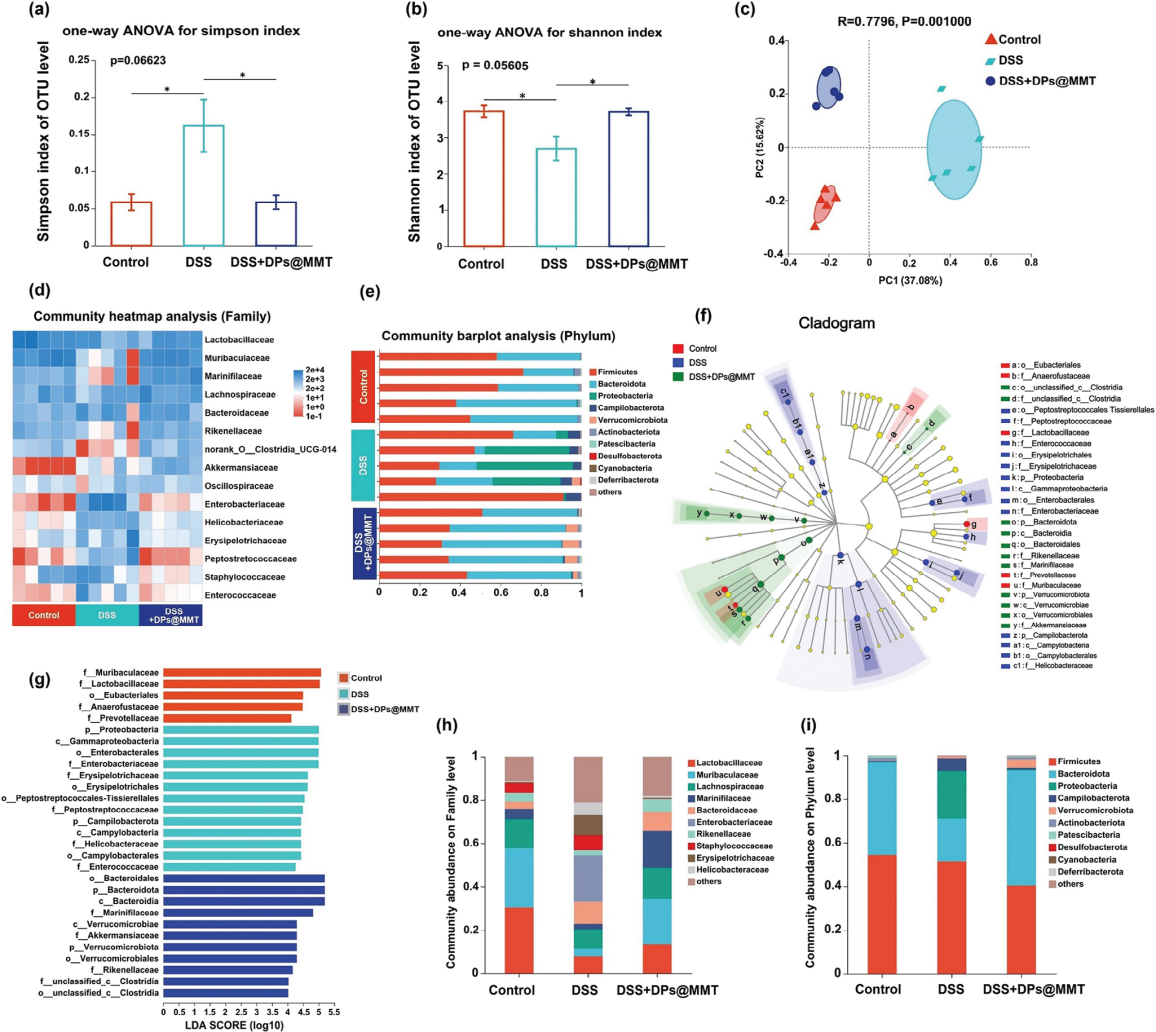
16S rRNA sequencing analysis of the gut microbiome regulated by DPs@MMT in DSS‐induced colitis mice. a,b) *α*‐diversity analysis by Simpson index and Shannon index; c) *β*‐diversity analysis by Principal co‐ordinates analysis; d) Heatmap of microbial composition at the family level; e) Histogram of microbial community composition at the phylum level; f) A cladogram diagram showing the microbial species with significant differences; g) LDA identification of the most abundant genus in different groups; h‐i) Significantly altered microbial communities at the family and phylum level; in Figure a,b: DSS group versus other groups (*n* = 5), *p < 0.05.

Gut flora, a crucial component of the intestinal mucosal barrier, plays a vital role in maintaining intestinal health.^[^ ^68^, ^69^ ^]^ Dysbiosis, or imbalances in the gut microbiota, is linked to various conditions including IBD and irritable bowel syndrome.^[^ ^70^ ^]^ Given the potent antioxidant, anti‐inflammatory, and antibacterial properties of DATS‐based H_2_S gas therapy,^[^ ^71^ ^]^ we investigated the regulatory impact of DPs@MMT on gut flora using a 16S ribosomal RNA (rRNA) gene sequencing assay. The Simpson and Shannon indices, indicative of community richness in the gut, revealed significant differences between the control and DSS groups. However, these differences were notably absent between the control and DPs@MMT groups, suggesting that DPs@MMT effectively restored gut flora diversity (**Figure** **8**a,b). *β*‐diversity analysis further confirmed that DPs@MMT successfully remodeled the gut microbiota composition (Figure 8c). The heat map and bar chart detailed the microbiome composition at the family and phylum levels across groups (Figure 8d,e). Linear discriminant analysis (LDA) effect size estimation identified key microbial groups differentially abundant across taxa (Figure 8f,g). Remarkably, DPs@MMT treatment significantly altered the microbiome composition at the family and phylum levels compared to the DSS group (Figure 8h,i), characterized by a marked reduction in harmful microbes like Proteobacteria and Enterobacteriaceae, and a substantial increase in beneficial bacteria such as Bacteriodota and Muribaculaceae. These beneficial microbes contribute to a healthy gut by inhibiting harmful microbes and regulating mucosal immune homeostasis. The microbiome profile of the DPs@MMT group closely resembled that of the control group, suggesting effective microbiome regulation. Interestingly, MMT, used for diarrhea relief, showed limited impact on gut microbiome modulation. The microbial community's *α*‐ and *β*‐diversity and composition at the family and phylum levels remained largely unchanged in the DSS+PDNs@MMT group compared to the DSS group, contrasting with the control group (Figure S13a–g, Supporting Information). This implies that the gut microbiome‐regulating effect of DPs@MMT is primarily attributed to DATS. In conclusion, DPs@MMT not only alleviates colitis symptoms but also positively influences the gut microbiome by enhancing its diversity and balance. This dual action highlights DPs@MMT's potential as a promising therapeutic for IBD, offering a unique strategy that combines direct treatment with microbiome modulation.

## 3 Conclusion

We have developed DPs@MMT, an innovative H_2_S‐releasing MMT nanoformulation, specifically designed for the treatment of IBD. This advanced nanoformulation excels in remodeling intestinal mucosal immune homeostasis, modulating gut microbiota, and repairing the mucosal barrier. Upon rectal administration, DPs@MMT preferentially binds to and covers the damaged intestinal mucosa, ensuring an efficient concentration of DPs at the IBD site for uptake by activated immune cells. Within these cells, DPs release H_2_S in response to glutathione, initiating a range of therapeutic effects. Extensive in vitro and in vivo studies have confirmed the multifunctional efficacy of DPs@MMT. It has been shown to effectively scavenge ROS, reduce inflammatory responses, repair the mucosal barrier, eliminate harmful microbes, and alleviate colitis symptoms. RNA sequencing has shed light on the mechanisms underlying these effects. DPs@MMT enhances antioxidant defense by promoting nuclear translocation of Nrf2 and inducing HO‐1 and other antioxidant enzymes. It also exhibits potent anti‐inflammatory properties by inhibiting critical signaling pathways, including p38/ERK MAPK, p65 NF‐κB, JAK‐STAT3, and HIF‐1‐induced glycolysis, while activating the PI3K‐AKT pathway. The effects of DPs@MMT extend to colonic mucosal barrier repair, which was achieved by protecting IECs from oxidative‐induced apoptosis, promoting IECs proliferation via the RAS/MAPK/AP‐1 pathway, and increasing the expression of TJPs. In addition, 16S rRNA sequencing revealed its critical role in gut microbiota remodeling, effectively reducing pathogenic bacteria and enhancing beneficial probiotics. In conclusion, DPs@MMT stands out as a versatile and comprehensive nanoformulation for the treatment of IBD. Its unique ability to simultaneously address oxidative stress, inflammation, mucosal barrier damage, and gut microbiota dysbiosis positions it as a novel and potentially transformative therapy in the field of IBD treatment.

## 4 Experimental Section

### Materials

OAS‐G3‐Lys was synthesized in the laboratory.^[^ ^72^, ^73^ ^]^ DSP was acquired from Beijing J&K Scientific Ltd. DATS was sourced from Shanghai Dibo Chemicals Technology Co., Ltd. Cy5.5‐NHS was procured from Solarbio. MMT was obtained from Energy Chemical Co., Ltd. LPS from *E. coli* O111:B4 was purchased from Sigma‐Aldrich. DCFH‐DA, WSP‐5, Cell Counting Kit‐8 (CCK‐8), and Live/Dead Cell Double Staining Kit were supplied from Shanghai Maokang Biotech. Co., Ltd. The total antioxidant capacity assay kit (ABTS method), SOD assay kit, GSH‐Px assay kit (DTNB method), and CAT kit were acquired from Suzhou Grace Biotechnology Co., Ltd. RiboAPO one‐step TUNEL apoptosis assay kit and Cell‐Light EdU Apollo 567 imaging kit were sourced from Guangzhou Ribobio. Rat IL‐1β, IL‐6, TNF‐α, IL‐4, IL‐10, p‐ERK, and p‐P38 ELISA kits were from Proteintech Group, Inc. The mouse phosphotylinositol 3 kinase (PI3K) ELISA kit was obtained from Jiangsu Enzyme Immune Industrial Co., Ltd. Hoechst 33 342 staining solution and H&E staining kit were purchased from Nanjing Keygen Biotech. Co., Ltd. Antibodies targeting ERK, p‐ERK, STAT3, HO‐1, GAPDH, and actin, as well as the antibody to p‐STAT3, were provided by Proteintech Group, Inc. and Abcam (USA), respectively. Antibodies against iNOS, CD86, and CD163 were from Bios. The Anti‐DNA/RNA oxidative damage antibody was procured from StressMarq Biosciences Inc. Dex‐p was sourced from Dalian Meilun Biotech Co., Ltd. *S. aureus* (ATCC25923), *E. coli* (ATCC25922), *C. rodentium* (ATCC51116), RAW264.7 mouse macrophages (SCSP‐5036), and Caco‐2 cells (TCHu146) were acquired from the American Type Culture Collection (ATCC).

### Preparation of DPs

To synthesis PDNs, a solution of N, N‐dimethylformamide (DMF) containing 20 mg of DSP was incrementally added to another DMF solution with 200 mg of OAS‐G3‐Lys. The reaction mixture was then maintained at room temperature for one day. Following this, the mixture underwent extensive dialysis against DMF and water, culminating in the collection of the final product after freeze‐drying. For integrating DATS into the PDNs, different quantities of DATS (50, 75, or 116 mg) were dissolved in methanol (MeOH). This DATS solution was then added dropwise to a MeOH solution containing 50 mg of PDNs. The combined solution was subjected to ultrasonication for 10 min and subsequently maintained at 37 °C for a day. After this incubation period, the mixture underwent extensive dialysis against water for another day, followed by lyophilization to yield DATS@PDNs, henceforth referred to as DPs. For labeling, a 50 µL Cy5.5‐NHS solution (1 mg mL^−1^) was combined with 2 mL of DPs (0.5 mg mL^−1^) and left to react at room temperature for 4 h. This was followed by exhaustive dialysis against ultrapure water to eliminate unbound dye, resulting in Cy5.5‐DPs. The particle size, zeta potential, and morphological characteristics of both PDNs and DPs were ascertained using dynamic light scattering (DLS, Nano ZS90) and TEM (JEM‐2100Plus). The amount of DATS loaded in the DPs and the efficacy of this loading were quantitatively evaluated using an elemental analyzer (EA, Elemental Vario EL).

### Preparation of DPs@MMT

For the exfoliation of MMT, 3 g of bulk MMT were initially immersed in 50 mL of sodium hydroxide solution, maintaining a pH range of 9 to 10. This suspension was then stirred vigorously for 24 h.^[^ ^36^ ^]^ Ultrasonication was employed to further exfoliate the suspension, aiding in the isolation of few‐layered or monolayer MMT.^[^ ^74^ ^]^ Following this, the suspension was subjected to centrifugation and subsequently freeze‐dried to acquire exfoliated MMT, characterized by its white, flaky solid appearance. In the next phase, 10 mL of a DPs solution (3 mg mL^−1^) was amalgamated with 10 mL of the previously prepared exfoliated MMT solution (60 mg mL^−1^). This combination was stirred rigorously. The reaction mixture was then allowed to settle at room temperature for 4 h. Post‐reaction, centrifugation was employed to separate unabsorbed DPs, and the resulting precipitate was either redispersed in water or lyophilized for future applications. The morphological characteristics of DPs@MMT was examined using a SEM (Zeiss Gemini 300). To determine the adsorption efficiency and content of DPs on MMT, a standard curve for Cy5.5‐DPs was initially established. Subsequently, Cy5.5‐DPs@MMT was prepared, and the supernatant was collected to measure the concentration of unabsorbed DPs. This data was then utilized to calculate both the adsorption efficiency and the content of DPs adsorbed onto the MMT surface.

### Specific Binding and Coverage of the Damaged Intestinal Mucosa

To ascertain the specific binding and coverage efficacy of DPs@MMT on damaged intestinal mucosa, mice with induced colitis were administered 0.2 mL of either Cy5.5‐DPs@MMT (30 mg mL^−1^) or Cy5.5‐DPs (1.5 mg mL^−1^) via rectal injection. 1 h post‐administration, the mice were euthanized, and their colon tissues were harvested for analysis. These tissues were subsequently fixed, stained using Hoechst 33 342, and examined under a confocal laser scanning microscope (CLSM, ZEISS LSM700, Germany). Additionally, ultra‐thin sections of the fixed colon tissues were prepared for detailed observation using TEM. Furthermore, the long‐term retention of DPs@MMT within the colonic tissue was monitored using an in vivo imaging system (IVIS, Lumina XRMS Series III, PerkinElmer).

### Dissociation and H_2_S Release Behavior of DPs@MMT

To evaluate the dissociation characteristics of DPs@MMT, it was incubated in simulated colonic fluid (pH 6.5) over varying durations. Subsequently, DLS was employed to measure changes in particle size distribution and PDI. Additionally, the in vitro H_2_S release profile of DPs@MMT was determined using a specialized H_2_S detection system. In this process, 2 mL of the DPs@MMT solution (2 mg mL^−1^, pH 6.5) was placed within a sealable cylindrical glass container alongside the H_2_S detector. To initiate the release of H_2_S, GSH solution at concentrations of 0, 2.0, 3.0, or 4.0 mm was injected into the DPs@MMT solution. The concentration of H_2_S (in ppm) was continuously monitored and recorded, allowing for the calculation of the amount of H_2_S released, as described in previous studies.^[^ ^75^ ^]^

### Cell Viability

RAW264.7 macrophages were cultured in a specialized medium (CM‐0190, Wuhan Pricella) at 37 °C in a 5% CO_2_ atmosphere. Similarly, Caco‐2 cells were maintained in their specific medium (CM‐0050, Wuhan Pricella) under the same conditions. Activated macrophages were prepared by stimulating RAW 264.7 cells with 5 µg mL^−1^ of LPS, while normal macrophages were RAW 264.7 cells without LPS treatment. For CCK‐8 assay, normal macrophages and Caco‐2 cells were initially seeded in 96‐well plates overnight. Subsequently, they were treated with DPs@MMT (2 mg mL^−1^) for an additional 24 hours, after which cell viability was assessed using the CCK‐8 method. The live/dead staining assay involved rinsing the cells, staining them, and observing them under an inverted fluorescence microscope (AxioObserver3, Zeiss). Cells treated with PBS, MMT at 2 mg mL^−1^, or DPs at 200 µg mL^−1^ served as control groups in these experiments.

### Intracellular H_2_S Imaging

To assess the uptake of DPs@MMT by cells, activated macrophages were initially cultured overnight, followed by treatment with Dulbecco's Modified Eagle Medium (DMEM) containing 2 mg mL^−1^ of Cy5.5‐DPs@MMT. After 2 hours, the cells were thoroughly rinsed and then imaged using CLSM. The fluorescence intensity of Cy5.5 within the cells was quantitatively analyzed using Image J software. To detect intracellular H_2_S release, WSP‐5 was employed.^[^ ^76^ ^]^ For this, activated macrophages were again cultured overnight and subsequently exposed to DMEM containing 2 mg mL^−1^ DPs@MMT. After a 4‐h incubation period, the culture medium was replaced with 300 µL of fresh DMEM containing 5 µM WSP‐5 and incubated for an additional 30 min. Post incubation, the cells were washed and visualized using CLSM. The fluorescence intensity of WSP‐5 in the cells was measured using a microplate reader.

### Antioxidant

Intracellular scavenging of ROS following H_2_S release was monitored using DCFH‐DA. Activated macrophages underwent prior treatment as outlined, followed by incubation with 300 µL of DMEM containing 5 µm DCFH‐DA for 30 min. Post‐incubation, the cells were washed and examined using CLSM. The fluorescence intensity of the resulting DCF was quantitatively measured using a microplate reader. The scavenging capacity of intracellular free radicals was measured using the ABTS assay. Post‐treatment with DPs@MMT, the activated macrophages were sonicated intermittently in an ice bath (300 W, 5 s/25 s cycles) for 30 min. The supernatant, obtained post‐centrifugation, had its protein concentration measured via a BCA protein assay kit. For the ABTS assay, 50 µL of this supernatant was combined with 950 µL of ABTS^+^ reagent and allowed to react for 6 minutes. The absorbance of the mixture at 734 nm was then measured using a UV spectrophotometer (Hitachi UH5700). The scavenging capacity for ABTS^+^ was computed as per the manufacturer's guidelines. The activities of CAT, SOD, and GSH‐Px were evaluated using their respective assay kits. Varying volumes of the supernatant were mixed with different types of test reagents following the manufacturer's instructions. The absorbance readings of these mixtures were then taken at 510 nm, 450 nm, and 412 nm for CAT, SOD, and GSH‐Px assays, respectively, using a microplate reader. The enzymatic activities of CAT, SOD, and GSH‐Px were calculated according to provided formulas and expressed as units per milligram of protein (U mg prot^−1^), micromoles per minute per milligram of protein (µmol min^−1^ mg prot^−1^), and nanomoles per minute per milligram of protein (nmol min^−1^ mg prot^−1^), respectively.

To assess the expression levels of p‐Nrf2 and HO‐1 following DPs@MMT treatment, activated macrophages treated with DPs@MMT were harvested for analysis. The expression levels of HO‐1 protein and its mRNA, as well as p‐Nrf2 protein, were quantified through WB and RT‐qPCR assays, respectively. For RT‐qPCR analysis, mRNA was extracted from the lysed cell samples. The RT‐qPCR process then followed, which involved reverse transcription of the mRNA, and subsequent amplification and quantification using a real‐time fluorescence quantitative PCR system. The specific primers utilized for this study are enumerated in Table S3 (Supporting Information). For WB analysis, protein extraction commenced with cell lysis in RIPA buffer containing protease inhibitors. The protein concentration was subsequently determined using a BCA protein assay kit. The proteins were then processed through a series of steps: electrophoresis for separation, membrane transfer, blocking, and incubation with primary antibodies targeting p‐Nrf2 and HO‐1. The proteins were visualized using an Enhanced Chemiluminescence (ECL) kit coupled with a gel imaging analysis system. Quantitative analysis of the protein bands was conducted using Image J software, with Nrf2 and β‐actin serving as internal controls for normalization. For IF staining of Nrf2 in activated macrophages, the cells were initially cultured in a medium containging DPs@MMT (2 mg mL^−1^) for 24 h, followed by stimulation with LPS (5 µg mL^−1^) for 100 min.^[^ ^77^ ^]^ Subsequently, the cells were washed, fixed, and stained with an anti‐Nrf2 antibody. After incubation with FITC‐labeled secondary antibodies and staining with F‐actin and DAPI, the cells were analyzed using a CLSM.

### Anti‐Inflammatory

After treating activated macrophages with DPs@MMT as previously described, the culture medium was collected. The levels of various inflammatory cytokines, namely IL‐1β, IL‐6, TNF‐α, IL‐4, IL‐10, and PI3K, were quantitatively analyzed using ELISA kits specific to each cytokine. The expression levels of key signaling proteins, specifically the phosphorylated and total forms of p38 MAPK (p‐p38, p38), ERK (p‐ERK, ERK), NF‐kB p65 (p‐p65, p65), and STAT3 (p‐STAT3, STAT3), were quantified using WB assays. Morphological examination of the treated macrophages was conducted using light microscopy. Parameters such as cell elongation ratio and synaptic number were quantified.^[^ ^78^ ^]^ Moreover, macrophages were immunolabeled with antibodies against iNOS and CD163 and observed under an inverted fluorescence microscope. The levels of mRNA and proteins for iNOS and CD163 were also quantified. For the flow cytometry analysis, the macrophages were initially washed and fixed. They were then incubated with either iNOS or CD206 antibody, followed by analysis using an ECKMAN flow cytometer (CytoFLEX).

### Restoration of Intestinal Mucosal Barrier

To evaluate the effects of DPs@MMT treatment on the anti‐apoptotic, pro‐proliferative, and wound‐healing capabilities of Caco‐2 cells, the culture medium was first harvested from activated macrophages treated with DPs@MMT. This medium was then used to culture Caco‐2 cells. In the anti‐apoptotic assay, Caco‐2 cells were seeded in 96‐well plates overnight and subsequently exposed to 200 µL of a freshly prepared medium, comprising a blend of 100 µL of the DPs@MMT‐treated activated macrophage culture medium and 100 µL of new Caco‐2 culture medium, for an additional 24 hours. Post‐treatment, the cells were fixed, permeabilized, and stained with TUNEL and DAPI solutions, followed by examination under an Axio Observer 3m inverted microscope from Zeiss. For the proliferation assessment, Caco‐2 cells were initially treated in the same manner and then incubated in 200 µL of fresh culture medium containing 10 mM EdU for an additional 6 h. These cells were then fixed, permeabilized, and stained with DAPI before being visualized using the same inverted microscope. In the wound healing experiment, Caco‐2 cells were cultured in three‐well Ibidi culture inserts within a 6‐well plate for 24 h at 37 °C. After removing the culture inserts to create a 0.5 mm scratch, the cells were incubated in 200 µL of fresh culture medium, a mix of 100 µL each of DPs@MMT‐treated activated macrophage culture medium and new Caco‐2 culture medium, for an extended period of 36 h. Following this, the cells were stained with crystal violet and observed under the inverted microscope. The fluorescence intensity of the images and the width of the scratch were quantified using ImageJ software.

### RNA Sequencing

For the RNA sequencing analysis, activated macrophages treated with DPs@MMT and Caco‐2 cells incubated in the culture medium of DPs@MMT‐treated macrophages were subjected to lysis using TRIZOL reagent for RNA extraction. The RNA's purity and concentration were accurately determined with a Nanodrop 2000 spectrophotometer, while its integrity was assessed using an Agilent 5300 Bioanalyzer. Only RNA samples that met the stringent quality criteria were advanced for library construction. These prepared RNA libraries underwent a thorough quality evaluation employing the Agilent 2100 Bioanalyzer and the Agilent High Sensitivity DNA Kit. In addition, qPCR was utilized to precisely measure the concentration of the validated libraries, ensuring their adequacy for sequencing purposes. The high‐quality RNA libraries were then sequenced on the Illumina NovaSeq 6000 platform, using a paired‐end 150 bp sequencing approach. The raw reads obtained from this sequencing were then refined into clean data using Fastp software, a critical step for ensuring data accuracy and reliability. Subsequently, the clean reads were aligned to the reference genome using the HISAT2 software.^[^ ^79^ ^]^ This step was crucial for accurate mapping and understanding of gene expression patterns. Finally, StringTie^[^ ^80^ ^]^ software was employed to assemble the mapped reads of each sample based on the reference genome, enabling the acquisition of raw counts.

The analysis of raw counts was conducted using R software (version 4.3.1), utilizing a suite of specific R packages for data processing and visualization. The normalization of raw counts was achieved through the Limma package, facilitating the generation of insightful principal component plots. DEGs was identified based on established criteria^[^ ^22^ ^]^ and this analysis was instrumental in producing informative volcano plots. The pheatmap package in R further enhanced the visualization, enabling the detailed representation of DEG expression via heatmaps. GO and KEGG pathway enrichment analyses were conducted using the clusterProfiler package.^[^ ^81^ ^]^ This analysis provided a clearer understanding of the underlying biological functions and pathways associated with the DEGs. The Z‐score metric was employed to determine the likelihood of a biological process, molecular function, or cellular component being inhibited (Z‐score < 0) or enhanced (Z‐score > 0). GSEA was also carried out to ascertain the significant enrichment of gene sets in relevant pathways. The p‐values, derived from the hypergeometric test, were rigorously adjusted for multiple testing using the Benjamini‐Hochberg method to ensure statistical reliability. Lastly, annotation of mouse and human genome information was accurately performed using the respective R packages “org.Mm.eg.db” and “org.Hs.eg.db”.

### Antimicrobial

The antimicrobial efficacy of DPs@MMT was assessed through colony counting and Live/Dead staining assays. *S. aureus*, *C. rodentium*, and *E. coli* were cultivated in lysogenic broth (LB) medium at 37 °C overnight. Post‐cultivation, the bacterial cells were isolated through centrifugation. Subsequently, 850 µL of each bacterial suspension (10^8^ CFU mL^−1^) was amalgamated with 150 µL of DPs@MMT (2 mg mL^−1^). This mixture was then incubated at 37 °C for 4 h. Following incubation, the bacterial suspensions were diluted, ranging from 10^6^ to 10^2^ CFU mL^−1^, and then evenly spread onto LB agar plates. These plates were further incubated at 37 °C for an additional 18 h. Post‐incubation, the colonies present on each plate were counted to determine the survival rate of the bacteria, offering a quantitative measure of the antimicrobial activity of DPs@MMT. For the Live/Dead staining assay, bacteria were first stained with a combination of SYTO‐9 (1 mm) and propidium iodide (PI) (1.5 mM) for 30 min. The stained bacterial samples were then examined under an inverted fluorescence microscope to visually assess the viability of the bacterial cells. Control groups for this experiment included bacteria treated with PBS and PDNs@MMT, which served as baseline comparisons for evaluating the antimicrobial effectiveness of DPs@MMT.

### Therapeutic Effect of DPs@MMT Against DSS‐Induced Colitis in Mice

This study's animal experiments received approval from the Animal Care and Use Committee at Wenzhou Medical University (protocol number: XMSQ2021‐0102). Female BALB/c mice, weighing between 20–25 grams, were procured from Beijing SPF Biotech Co., Ltd. and acclimatized for a duration of seven days prior to their inclusion in the experiment. The induction of colitis was achieved by administering 3% (w/v) DSS via drinking water over a period of one week. Subsequently, thirty mice were randomly divided into six groups (*n* = 5 per group) and continued to receive 3% (w/v) DSS in their drinking water for an additional nine days. Each mouse with colitis underwent a single rectal administration of 200 µL of either PBS, Dex‐p (75 µg mL^−1^), MMT (30 mg mL^−1^), DPs (1.5 mg mL^−1^), or DPs@MMT (30 mg mL^−1^) on days 3, 5, 7, and 9. The study closely monitored changes in body weight, stool consistency, and presence of blood in the stool on a daily basis. The DAI was calculated following the methodology outlined in previous research.^[^ ^27^ ^]^ The ROS scavenging capability of DPs@MMT was evaluated on day 11 by intraperitoneal injection of 80 µL L‐012 probe solution (6 µg mL^−1^), followed by capturing luminescence images of the inflamed intestines using an in vivo imaging system. Post‐experiment, all mice were euthanized, and their colonic tissues were collected for measurement of colon length. Histological analysis involved rinsing, fixing, embedding, sectioning, and H&E staining of colon segments and vital organs. The histological scoring was conducted as per the methods reported previously.^[^ ^82^ ^]^ To determine the anti‐inflammatory effects of DPs@MMT in vivo, colon segments ere homogenized, centrifuged, and levels of IL‐6, IL‐1β, and TNF‐α in the supernatants were measured using ELISA kits. Immunofluorescence staining involved sectioning and labeling of colon segments with antibodies against HO‐1, 8‐OHdG, CD86, and CD163, followed by visualization under an inverted fluorescence microscope. Orbital blood samples were collected for hematological analysis, which was conducted at Servicebio Biotech Co., Ltd., Wuhan.

### Microbiome Analysis

The 16S rRNA gene sequencing assay began with the surgical collection of fresh feces from four distinct groups of mice: a healthy control group, a set with DSS‐induced colitis receiving no treatment, and two other groups with DSS‐induced colitis, one treated with DPs@MMT and the other with PDNs@MMT. These samples were immediately frozen on dry ice and then stored at an ultra‐low temperature of −80 °C. Total genomic DNA extraction from these samples was carried out using the E.Z.N.A. soil DNA kit. The quality and concentration of DNA were meticulously assessed using agarose gel electrophoresis and a NanoDrop 2000 spectrophotometer, respectively. Amplification of the V3‐V4 region of the 16S rRNA gene was conducted using the primer pairs 338F and 806R, on an ABI GeneAmp 9700 PCR thermocycler. Subsequent analysis of the microbiome composition was performed using the Illumina MiSeq sequencing platform, adhering to the standard protocol provided by Majorbio Bio‐Pharm Technology Ltd. Operational Taxonomic Units (OTUs) were clustered with a 97% similarity threshold using UPARSE software (version 7.1). The taxonomy of each representative sequence from these OTUs was examined using the RDP Classifier version 2.2, against the Silva database (Release132/16s bacteria), with a confidence level set at 0.7. Comprehensive data analysis was executed on the Majorbio Biocloud platform to gain insights into the microbial community structure and diversity in each sample group.

### Statistical

Results are presented as the mean ± standard deviation (SD). All reported data are derived from a minimum of three independent experiments conducted in parallel. Statistical evaluations across all studies were conducted using one‐way analysis of variance (ANOVA). Data analysis was performed utilizing Prism software (version 9.0, GraphPad, La Jolla, CA, USA). Statistical significance was determined based on *p*‐values, with *p* < 0.05 indicating significance. The specific levels of significance are denoted as follows: * *p* < 0.05, ** *p* ≤ 0.01, *** *p* ≤ 0.001. A notation of “ns” represents a lack of statistical significance.

## Conflict of Interest

The authors declare no conflict of interest.

## Supporting information

Supporting Information

Supporting Information

## Data Availability

The data that support the findings of this study are available in the supplementary material of this article.
