# Correction to “H_2_S‐Releasing Versatile Montmorillonite Nanoformulation Trilogically Renovates the Gut Microenvironment for Inflammatory Bowel Disease Modulation”

**DOI:** 10.1002/advs.75007

**Published:** 2026-03-27

Ting Jin, Hongyang Lu, Qiang Zhou, Dongfan Chen, Youyun Zeng, Jiayi Shi, Yanmei Zhang, Xianwen Wang^*^, Xinkun Shen^*^, and Xiaojun Cai^*^

T. Jin, H. Lu, D. Chen, Y. Zeng, J. Shi, Y. Zhang, X. Cai

School and Hospital of Stomatology, Wenzhou Medical University, Wenzhou 325027, China

E‐mail: cxj520118@wmu.edu.cn; cxj520118@njtech.edu.cn

Q. Zhou, X. Shen

Department of Otolaryngology, Ruian People's Hospital, The Third Affiliated Hospital of Wenzhou Medical University, Wenzhou 325016, China

E‐mail: shenxinkun123@wmu.edu.cn

X. Wang

School of Biomedical Engineering, Research and Engineering Center of Biomedical Materials, Anhui Medical University, Hefei 230032, China

E‐mail: xianwenwang@ahmu.edu.cn

Jin, et al. H_2_S‐Releasing Versatile Montmorillonite Nanoformulation Trilogically Renovates the Gut Microenvironment for Inflammatory Bowel Disease Modulation. Advanced Science. 2024 Apr;11(14):2308092.

https://doi.org/10.1002/advs.202308092

After a careful post‐publication self‐review, we identified a few inadvertent errors resulting from figure assembly inaccuracies. Specifically, the representative images for the LPS group in Figure 2c, for the DPs group in Figure 4t, and for the DPs@MMT group in Figures 5a and 7b do not correspond to the correct groups.

In Figure 2c, the representative image for the LPS group was incorrect.


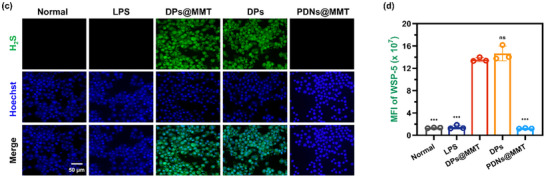


Corrected Figure 2c is shown below:


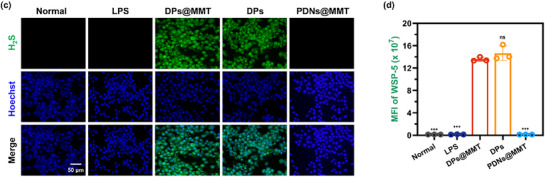


In Figure 4t, the representative image for the DPs group was incorrect.


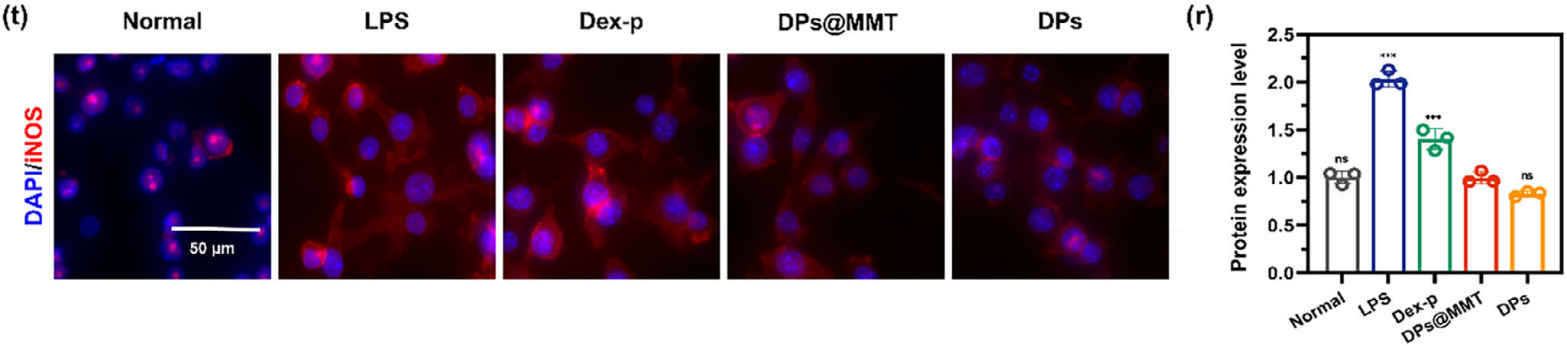


Corrected Figure 4t is shown below:


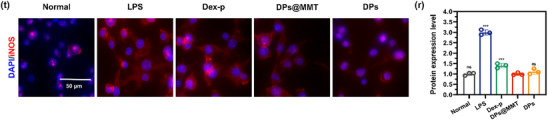


In Figure 5a, the representative image for the DPs@MMT group was incorrect.


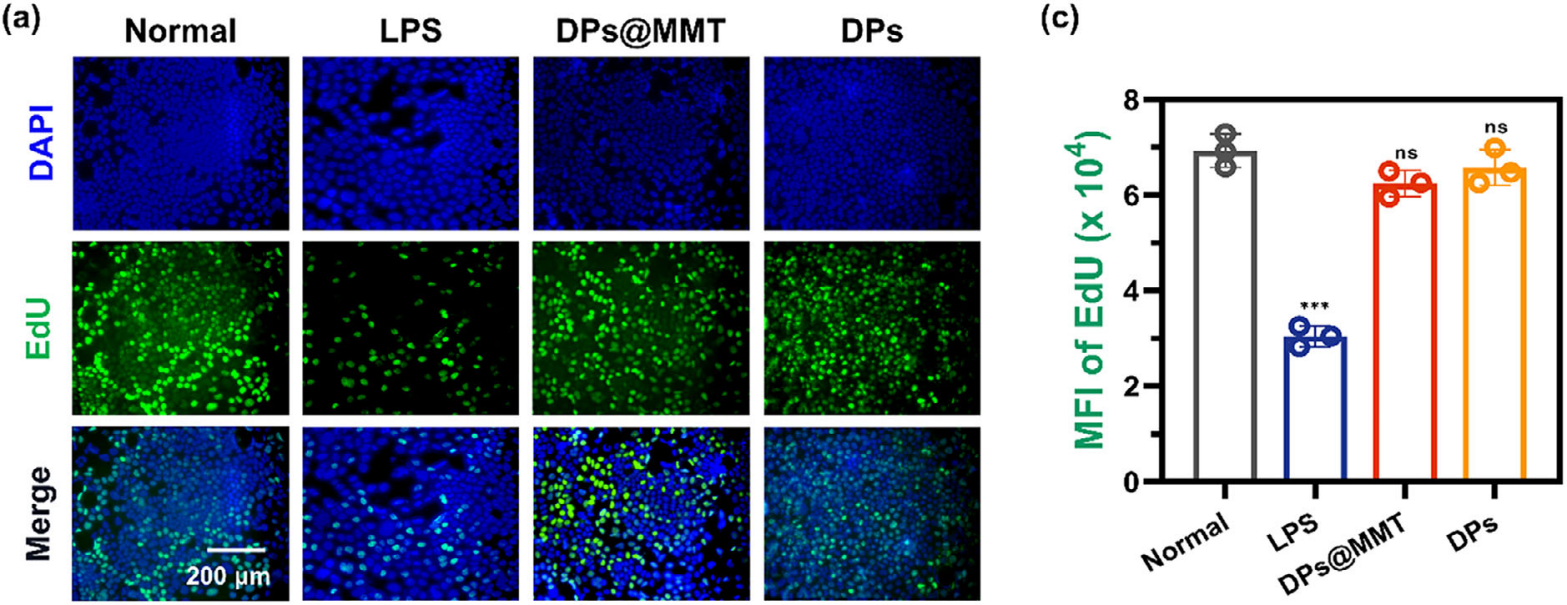


Corrected Figure 5a is shown below:


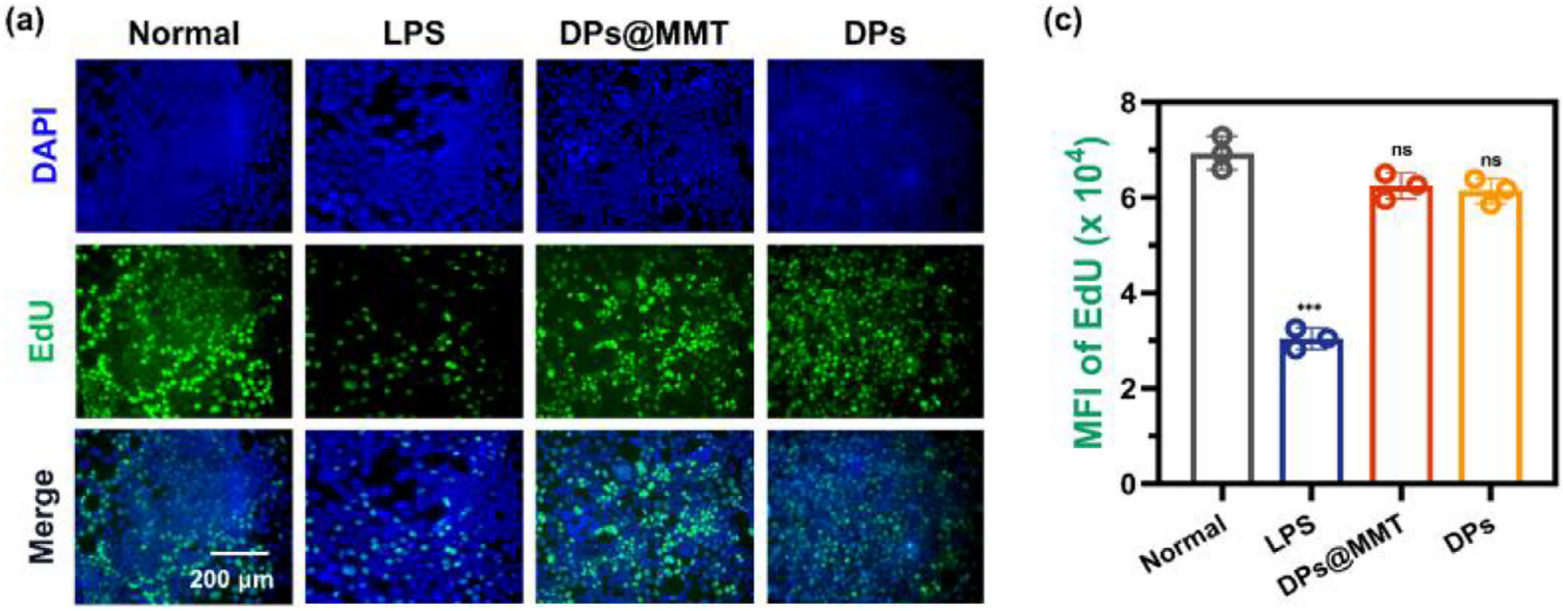


In Figure 7b, the representative image for the DPs@MMT group was incorrect.


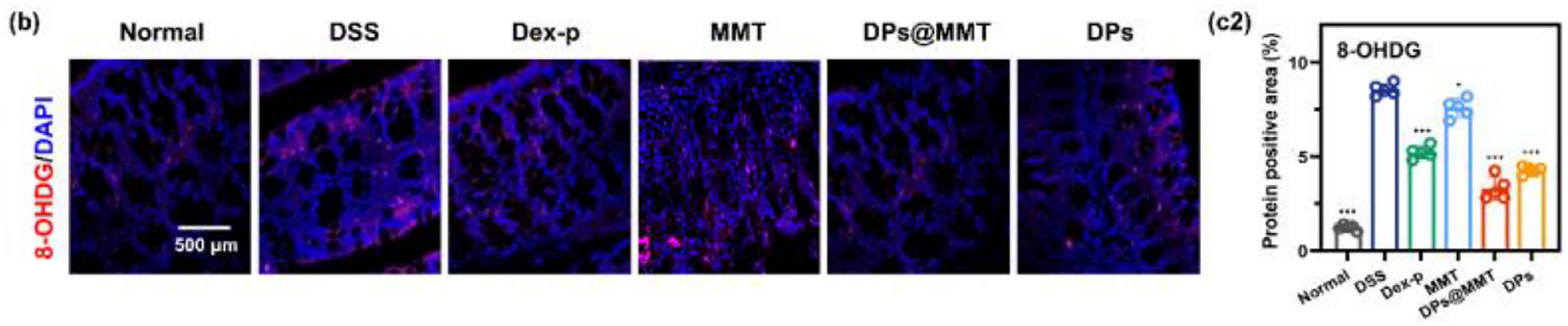


Corrected Figure 7b is shown below:


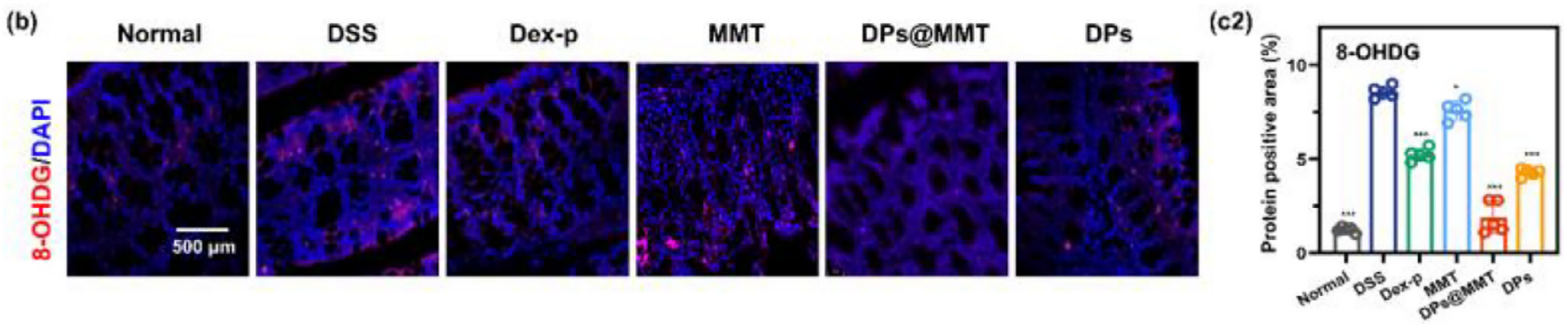


These corrections do not affect the overall findings and conclusions of the paper.

We sincerely apologize for these errors.

