# Supplementary material for: H2S‐Releasing Versatile Montmorillonite Nanoformulation Trilogically Renovates the Gut Microenvironment for Inflammatory Bowel Disease Modulation
Source: Adv Sci (Weinh). 2024 Feb 2;11(14):2308092. doi: 10.1002/advs.202308092 (PMC11005690; doi:10.1002/advs.202308092)
Supplement: Supplementary file 1 — Supporting Information [file ADVS-11-2308092-s002.pdf]

ADVANCED SCIENCE
Open Access

Supporting Information

for *Adv. Sci.*, DOI 10.1002/advs.202308092

H_2S-Releasing Versatile Montmorillonite Nanoformulation Trilogically Renovates the Gut Microenvironment for Inflammatory Bowel Disease Modulation

Ting Jin, Hongyang Lu, Qiang Zhou, Dongfan Chen, Youyun Zeng, Jiayi Shi, Yanmei Zhang, Xianwen Wang, Xinkun Shen* and Xiaojun Cai**

Supporting Information

H_2S-Releasing Versatile Montmorillonite Nanoformulation Trilogically Renovates the Gut Microenvironment for Inflammatory Bowel Disease modulation

Ting Jin, Hongyang Lu, Qiang Zhou, Dongfan Chen, Youyun Zeng, Jiayi Shi, Yanmei Zhang, Xianwen Wang, Xinkun Shen*, Xiaojun Cai**

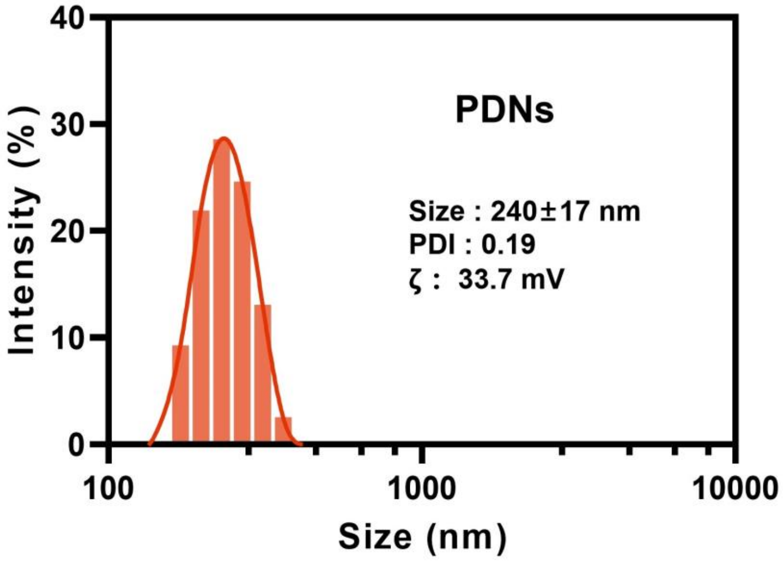

Figure S1. The particle size distribution and ζ-potential of PDNs.

Table S1. The loading content and efficacy of DATS in DPs with different feed ratios of DATS/PDNs.

DATS/PDNs	Loading content (%)	Loading efficacy (%)
40%	11.1	22.2
50%	23.5	39.1
60%	30.1	43.0

Table S2. Particle size and ζ-potential and adsorption efficiency and adsorption content of DPs@MMT with different feed ratios of DPs/MMT.

DPs/MMT	Size (nm)	PDI	ζ-potential (mV)	Adsorption efficiency (%)	Adsorption content (%)
1:25	594.6	0.25	-28.5	91.49	9.15
1:20	489.2	0.19	-26.2	93.85	4.69
1:10	497.4	0.16	10	95.36	3.81

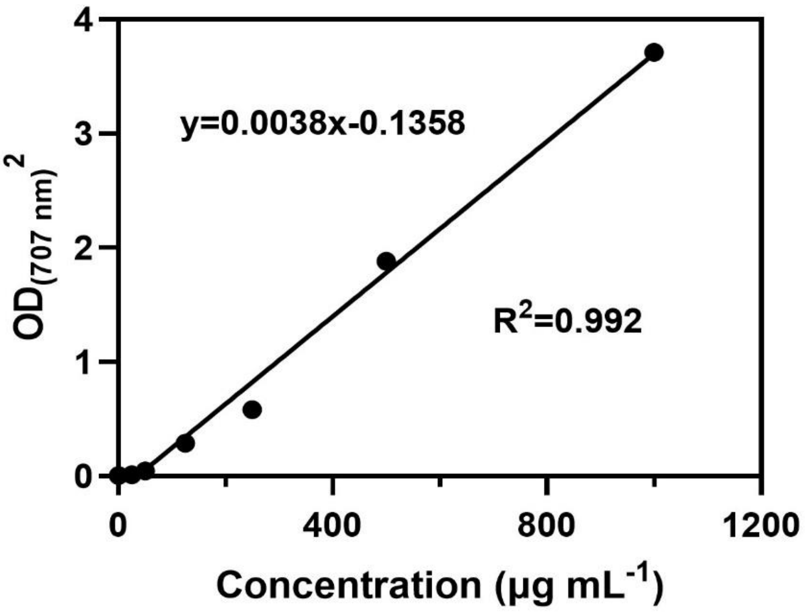

Figure S2. The standard curve of Cy5.5-labeled DPs.

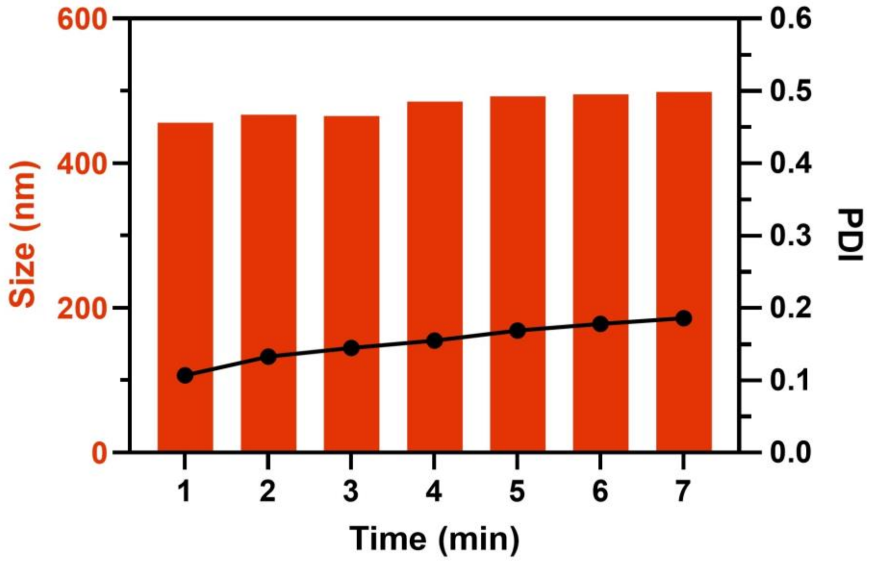

Figure S3. Particle size and PDI variation of DPs@MMT in PBS solution (pH=7.4) for 7 days.

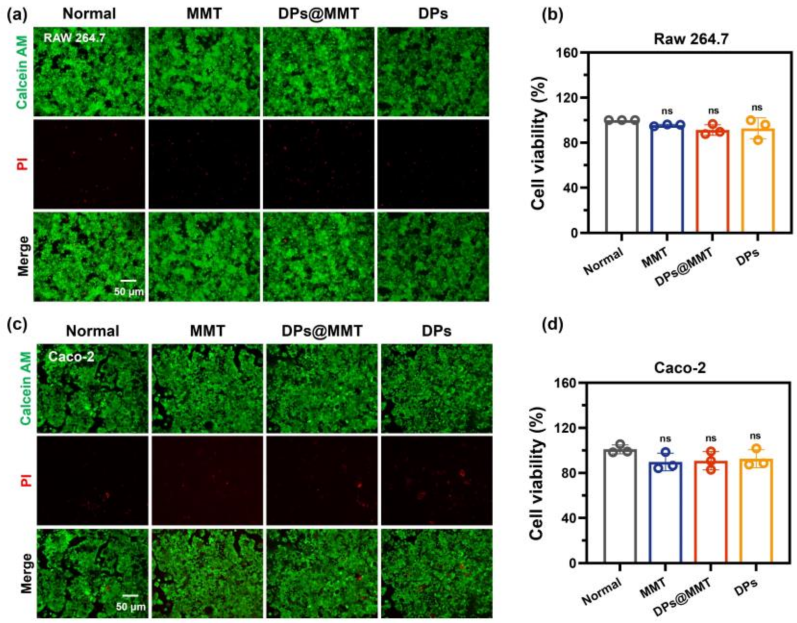

Figure S4. a&c) Live/dead staining and b&d) Cytotoxicity of Raw 264.7 macrophages and Caco-2 cells after different treatments. Data are presented as mean ± SD, n = 3, * $p < 0.05$, ** $p \leq 0.01$, *** $p \leq 0.001$, ns: no significance. All statistical analysis compare between normal group and other groups.

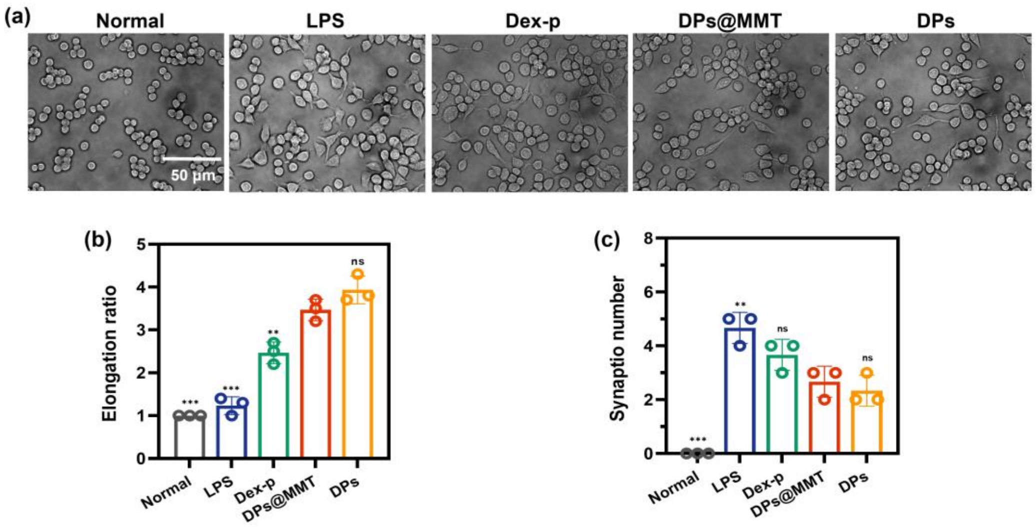

Figure S5. a) The macrophage polarization profiles and the corresponding b) elongation ratio and c) synapse number of activated macrophages after different treatments. Data are presented as mean ± SD, n = 3, * p <0.05, ** p ≤0.01, *** p ≤ 0.001, ns: no significance. All statistical analysis compare between DPs@MMT group and other groups.

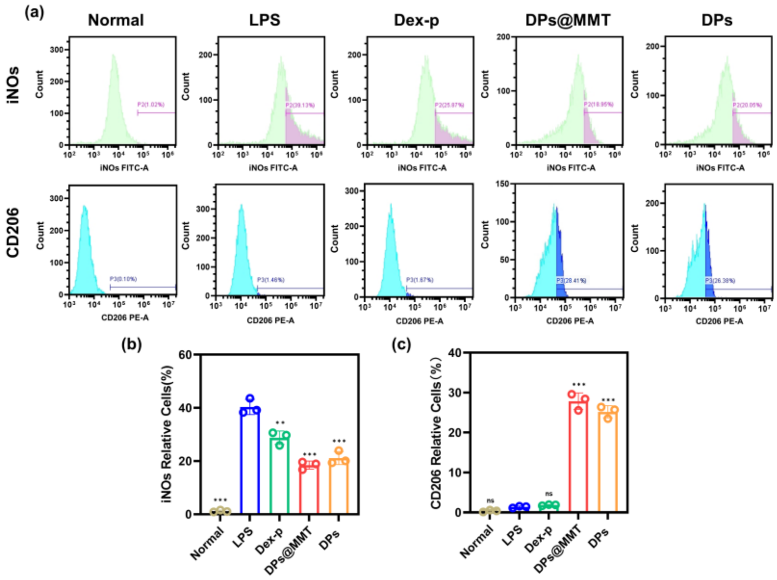

Figure S6. a) Flow cytometry analysis of the iNOS-positive M1 macrophages and CD206-positive M2 macrophages. b) iNOs and c) CD206 relative Cells (%) of activaed macrophage after receiving various treatments. Data are presented as mean ± SD, n = 3, * p <0.05, ** p ≤0.01, *** p ≤0.001, ns: no significance. All statistical analysis compare between DPs@MMT group and other groups.

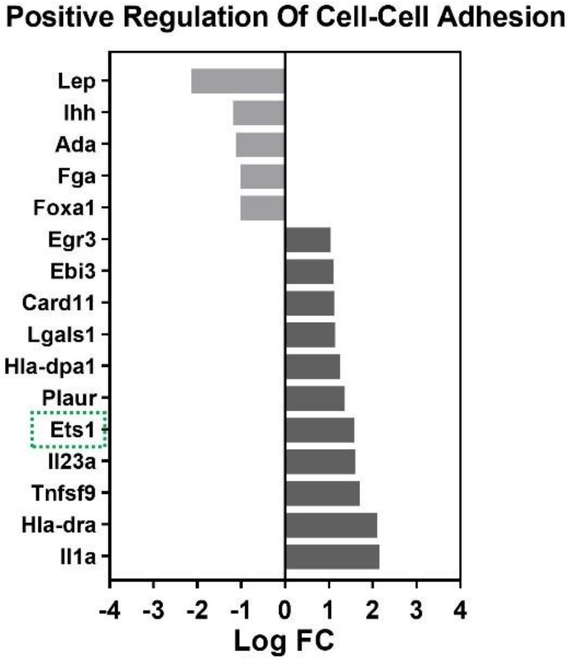

Figure S7. The expression of DEGs in Cell-Cell adhesion related signalling pathway.

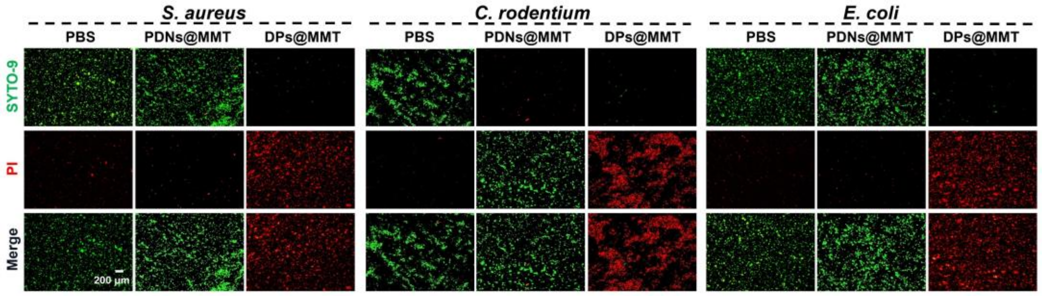

Figure S8. The Live/dead staining of *S. aureus*, *C. rodentium*, and *E.coli* after different treatments.

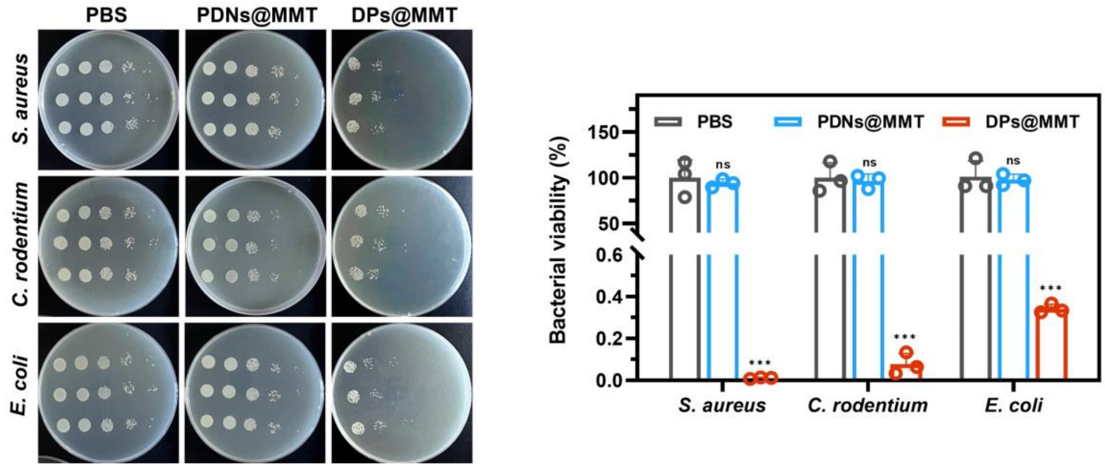

Figure S9. Representative images of plate samples and the survival rate of *S. aureus*, *C. rodentium* and *E. coli* after different treatments. Data are presented as mean ± SD, n = 3, * $p < 0.05$, ** $p \leq 0.01$, *** $p \leq 0.001$, ns: no significance. Statistical analysis compare between PBS group and other groups.

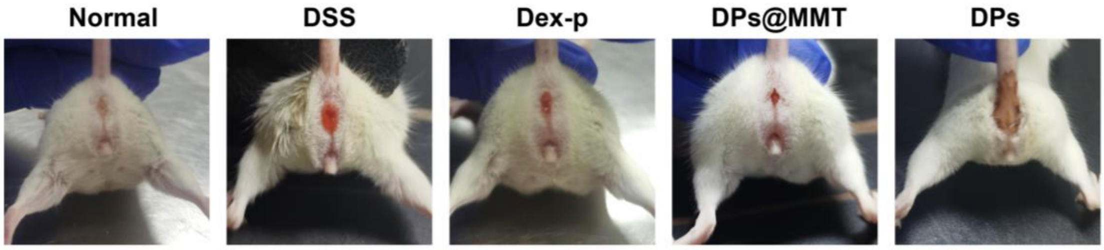

Figure S10. Occult blood phenomena in colitis mice after different treatments on day 11.

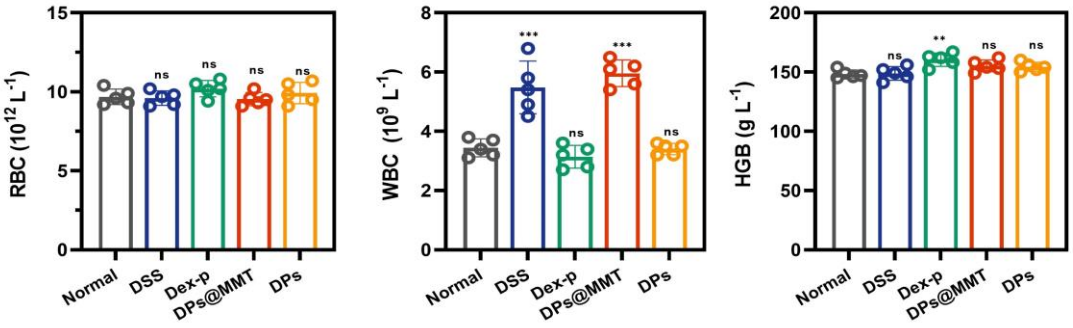

Figure S11. The red blood cell (RBC), white blood cell (WBC) and haemoglobin (HGB) counts of colitis mice after different treatments on day 11. Data are presented as mean ± SD, n = 5, * $p < 0.05$, ** $p \leq 0.01$, *** $p \leq 0.001$, ns: no significance. All statistical analysis compare between normal group and other groups.

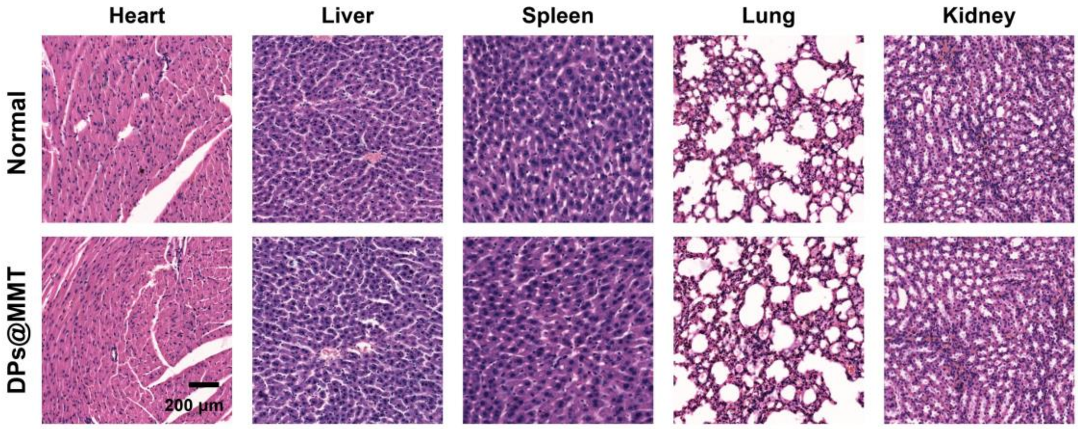

Figure S12. H&E staining of heart, liver, spleen, lung, and kidney of normal and DPs@MMT groups on day 11.

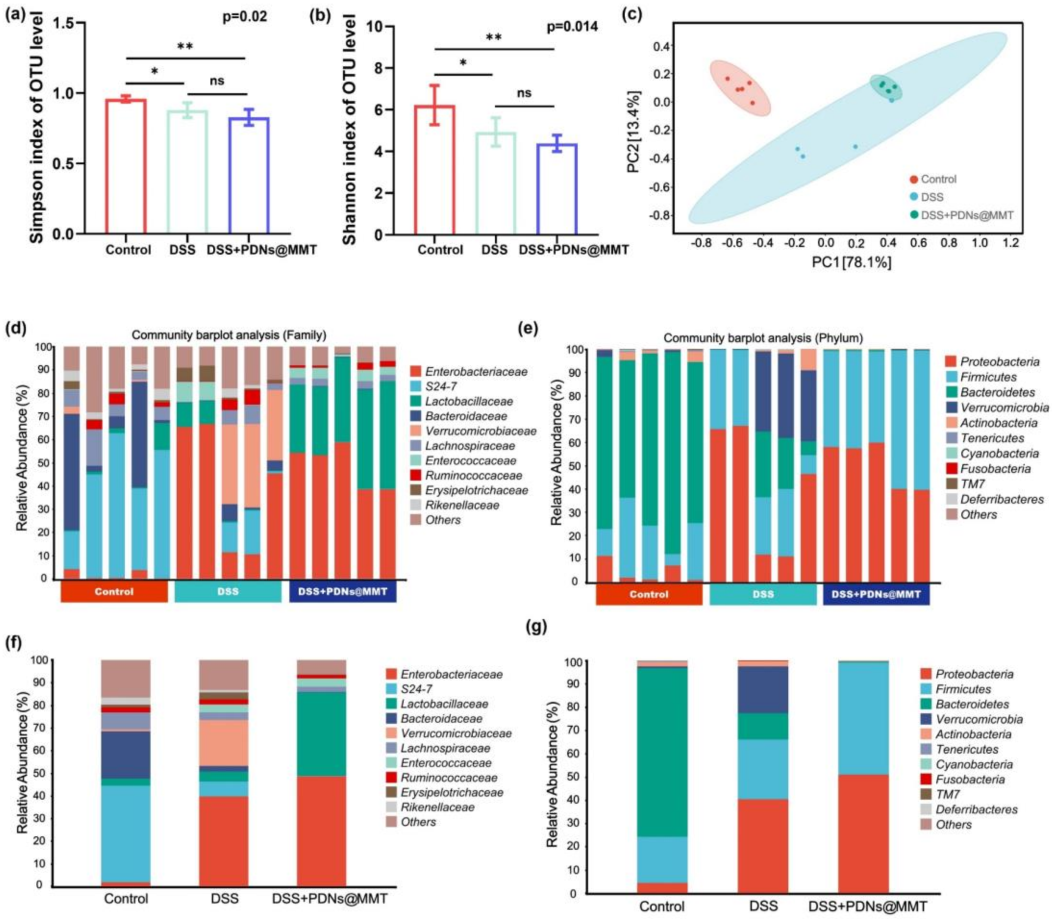

Figure S13. 16S sequencing analysis of gut microbiota regulated by PDNs@MMT. a) Simpson and b) Shannon index of the observed OUT shows the α-diversity of the microbial community; c) PCA shows the β-diversity of the gut microbiome. Each dot represents one mouse (n = 5); d,e) Community histogram shows the microbial compositional profiling at the family level and phylum level. Each row represents one mouse (n = 5). f,g) Relative abundance of microbiota communities that were significantly altered at the family and phylum level.

Table S3. The Primer sequence of genes used in the study.

Gene	Forward primer sequence (5'-3')	Reverse primer sequence (3'-5')
CD163	CTGGCGGGTGGAAAACA	CAGCCGTTACTGCACACTG
iNOS	CGCTTGGGTCTTGTTCACT	TCTTCAGGTCACTTGGTA
HO-1	AAGACTGCGTTCCTGCTCAAC	AAAGCCCTACAGCAACTGTCG
