# Supplementary material for: H2S‐Releasing Versatile Montmorillonite Nanoformulation Trilogically Renovates the Gut Microenvironment for Inflammatory Bowel Disease Modulation
Source: Adv Sci (Weinh). 2024 Feb 2;11(14):2308092. doi: 10.1002/advs.202308092 (PMC11005690; doi:10.1002/advs.202308092)
Supplement: Supplementary file 2 — Supporting Information [file ADVS-11-2308092-s001.zip › Supporting information/Supporting information 2. R script.doc]

**###设置环境变量以及镜像**

**rm(list = ls())**

**options()$repos**

**options()$BioC_mirror**

**#options(BioC_mirror="https://mirrors.ustc.edu.cn/bioc/")**

**options(BioC_mirror="http://mirrors.tuna.tsinghua.edu.cn/bioconductor/")**

**options("repos" = c(CRAN="https://mirrors.tuna.tsinghua.edu.cn/CRAN/"))**

**options()$repos**

**options()$BioC_mirror**

**###包的安装与使用**

**if (!requireNamespace("BiocManager", quietly = TRUE))**

**install.packages("BiocManager")**

**library(ggplot2)**

**library(limma)**

**library(pheatmap)**

**library(ggsci)**

**library(dplyr)**

**lapply(c('clusterProfiler','enrichplot','patchwork'), function(x) {library(x, character.only = T)})**

**library ("org.Mm.eg.db")**

**library(patchwork)**

**library(WGCNA)**

**library(GSEABase)**

**library(GSVA)**

**###路径切换及文件导入**

setwd("F://R//new")

rt=read.table("matrix.txt",sep="\t",header=T,check.names=F)

rt[rt==0]<-NA

rt=na.omit(rt)##去除所有含NA的行

rt=as.matrix(rt)

rownames(rt)=rt[,1]

exp=rt[,2:ncol(rt)]

dimnames=list(rownames(exp),colnames(exp))

rt=matrix(as.numeric(as.matrix(exp)),nrow=nrow(exp),dimnames=dimnames)

rt=avereps(rt)

rt1=normalizeBetweenArrays(as.matrix(rt))

####差异分析

data=rt1

#data=rt

####分组

afcon=4

conData=data[,as.vector(colnames(data)[1:afcon])]

aftreat=afcon+1

treatData=data[,as.vector(colnames(data)[aftreat:ncol(data)])]

rt=cbind(conData,treatData)

conNum=ncol(conData)

treatNum=ncol(treatData)

group=c(rep("con",4),rep("treat",4))

design <- model.matrix(~group)

y <- DGEList(counts=data,group=group)

y <- calcNormFactors(y)

y <- estimateCommonDisp(y)

y <- estimateTagwiseDisp(y)

et <- exactTest(y,pair = c("con","treat"))

DIFF<-topTags(et,n=10000000)#算FDR

DIFF<-DIFF$table

allDiff<-DIFF

write.table(allDiff,file=paste0("1.","alldiff_change.xls"),sep="\t",quote=F,col.names=T)

write.table(allDIFF,file=paste0("2.","martix_exchange.xls"),sep="\t",quote=F,col.names=T)

cut_off_pvalue = 0.05

cut_off_logFC = 1

allDiff$change = ifelse(allDiff$FDR < cut_off_pvalue & abs(allDiff$logFC) >= cut_off_logFC,

ifelse(allDiff$logFC> cut_off_logFC ,'Up','Down'),

'Stable')

library(ggplot2)

pdf(file="Volcano.pdf", height=10,width=12)

ggplot(

allDiff,

aes(x = logFC,

y = -log10(PValue),

colour=change)) +

geom_point(alpha=0.9, size=4) +

scale_color_manual(values=c("#016DDB","grey","#E43401"))+

\# 辅助线

geom_vline(xintercept=c(-1,1),lty=4,col="black",lwd=0.8) +

geom_hline(yintercept = -log10(cut_off_pvalue),lty=4,col="black",lwd=0.8) +

\# 坐标轴

labs(x="log2(fold change)",

y="-log10 (FDR)")+

theme_bw()+

\# 图例

theme(plot.title = element_text(hjust = 0.5),

legend.position="right",

legend.title = element_blank()

)

dev.off()

###GSEA分析

allDIFF<-read.table("allDIFF.txt",sep="\t",header=T,check.names=F)

gene.df <- bitr(allDIFF$SYMBOL,

fromType = "SYMBOL",

toType = c("ENTREZID"),

OrgDb = org.Mm.eg.db)

Enrich <- gene.df %>%

inner_join(allDIFF, by = "SYMBOL")

head(Enrich)

Enrichment <- Enrich %>%

as.data.frame()%>%

dplyr::select(ENTREZID,logFC)

Enrichment <- Enrichment[!duplicated(Enrichment[,1]),]

geneList <- Enrichment$logFC

as.data.frame(geneList)

names(geneList) <- Enrichment$ENTREZID

geneList <-sort(geneList,decreasing = T)

GSEA1 <- gseKEGG(geneList	= geneList,

organism	= 'mmu',

nPerm	= 10000,

minGSSize	= 1,#刷选最小

maxGSSize	= 10000,#刷选最大

pvalueCutoff = 0.05,

pAdjustMethod = "none" )

View(GSEA1@result)

write.table(GSEA1@result,file=paste0("1.","GSEA.xls"),sep="\t",quote=F,col.names=T)

kure<-GSEA1@result

View(kure)

GS<-GSEA1@geneSets

View(GS)

a<-GS$mmu04620

PI3K_symbol <- bitr(a,

fromType = "ENTREZID",

toType = c("SYMBOL"),

OrgDb = org.Mm.eg.db)

View(PI3K_symbol)

PI3K_symbol<-PI3K_symbol$SYMBOL

PI3K_symbol<-as.data.frame(PI3K_symbol)

PI3K_symbolexp<-allDIFF[allDIFF$SYMBOL %in% PI3K_symbol$PI3K_symbol, ]###差异分析

PI3K_symbolexpress<-rt[rt$SYMBOL %in% PI3K_symbol$PI3K_symbol, ]###表达矩阵

rownames(PI3K_symbolexp)<-NULL

rownames(PI3K_symbolexpress)<-NULL

rownames(PI3K_symbolexpress)=PI3K_symbolexpress[,1]

exp=PI3K_symbolexpress[,2:ncol(PI3K_symbolexpress)]

dimnames=list(rownames(exp),colnames(exp))

PI3K_symbolexpress=matrix(as.numeric(as.matrix(exp)),nrow=nrow(exp),dimnames=dimnames)

PI3K_symbolexpress=avereps(PI3K_symbolexpress)

rownames(PI3K_symbolexp)=PI3K_symbolexp[,1]

##########通路绘制

BiocManager::install("pathview")

library(pathview)

gene1.df <- bitr(PI3K_symbolexp$SYMBOL,

fromType = "SYMBOL",

toType = c("ENTREZID"),

OrgDb = org.Mm.eg.db)

Enrich1 <- gene1.df %>%

inner_join(PI3K_symbolexp, by = "SYMBOL")

head(Enrich1)

Enrichment1 <- Enrich1 %>%

as.data.frame()%>%

dplyr::select(SYMBOL,ENTREZID,logFC)

Enrichment1 <- Enrichment1[!duplicated(Enrichment1[,1]),]

gene <- Enrichment1$ENTREZID

gene_data<-Enrich1 %>%

as.data.frame()%>%

dplyr::select(ENTREZID,logFC)

rownames(gene_data)<-gene_data[,1]

gene_data$ENTREZID<-NULL

#KEGG view: gene data only

pv.out <- pathview(gene.data = gene_data,

pathway.id = "04620",

species = "mmu",

out.suffix ="toll", #输出文件的后缀

kegg.native = T, #输出的结果图为原始的KEGG pathway图,图片格式为".png"

gene.idtype = "ENTREZID",

limit = list(gene = 2, cpd = 2))

write.table(PI3K_symbolexp,file=paste0("1.","HIF.xls"),sep="\t",quote=F,col.names=T)

\#####

pdf(file="MAPK.pdf", height=8,width=12)

gseaplot2(GSEA1,

title = "MAPK", #设置标题

"hsa04010", #绘制hsa通路的结果，通路名称与编号对应

color="black", #线条颜色

base_size = 20, #基础字体的大小

subplots = 1:3,

pvalue_table = F) # 显示p值

dev.off()

##KEGG and GO

DIFF<-read.table("DIFF.txt",sep="\t",header=T,check.names=F)

gene.df <- bitr(DIFF$SYMBOL,

fromType = "SYMBOL",

toType = c("ENTREZID"),

OrgDb = org.Mm.eg.db)

Enrich <- gene.df %>%

inner_join(DIFF, by = "SYMBOL")

Enrichment <- Enrich %>%

as.data.frame()%>%

dplyr::select(SYMBOL,ENTREZID,logFC)

Enrichment <- Enrichment[!duplicated(Enrichment[,1]),]

gene <- Enrichment$ENTREZID

#GO

ego <- enrichGO(gene = gene,

OrgDb = org.Mm.eg.db, #

pvalueCutoff =0.05,

qvalueCutoff = 0.05,

ont="all",

readable =T)

write.table(ego@result,file=paste0("1.","GO.xls"),sep="\t",quote=F,col.names=T)

install.packages("GOplot")

#install_github(''wencke/wencke.github.io'')

###########加载

library(GOplot)

data(EC)

head(EC$david)#查看数据结构

head(EC$genelist) #查看数据结构

circ <- circle_dat(EC$david, EC$genelist)

allDIFF<-read.table("allDIFF.txt",sep="\t",header=T,check.names=F)

Go<-read.table("Go.txt",sep="\t",header=T,check.names=F)

circ<- circle_dat(Go, allDIFF)

###泡泡图

pdf(file="GO0.pdf", height=10,width=23)

reduced_circ <- reduce_overlap(circ, overlap = 0.75)

GOBubble(reduced_circ, labels = 1.63)

dev.off()

write.table(ego,file=paste0("1.","GO.xls"),sep="\t",quote=F,col.names=T)

head(ego)

#KEGG分析

KEGG1 <- enrichKEGG(gene = gene,

organism = "mmu",

pvalueCutoff =1,

qvalueCutoff =1)

write.table(KEGG1,file=paste0("1.","KEGGall.xls"),sep="\t",quote=F,col.names=T)

dev.off()

KK<-KEGG1@geneSets

GS<-KK$hsa04010

JAK_STAT <- bitr(GS,

fromType = "ENTREZID",

toType = c("SYMBOL"),

OrgDb = org.Hs.eg.db)

JAK_STAT<-JAK_STAT$SYMBOL

JAK_STAT<-as.data.frame(JAK_STAT)

JAK_STATexp<-DIFF[DIFF$SYMBOL %in% JAK_STAT$JAK_STAT, ]###差异分析

write.table(JAK_STATexp,file=paste0("1.","MAPKgene.xls"),sep="\t",quote=F,col.names=T)

head(kk)

library(GOplot)

data(EC)

head(EC$david)#查看数据结构

head(EC$genelist) #查看数据结构

circ <- circle_dat(EC$david, EC$genelist)

allDIFF<-read.table("DIFF.txt",sep="\t",header=T,check.names=F)

Go<-read.table("Go.txt",sep="\t",header=T,check.names=F)

circ<- circle_dat(Go, allDIFF)

###泡泡图

pdf(file="1.GO1.pdf", height=10,width=23)

reduced_circ <- reduce_overlap(circ, overlap = 0.75)

GOBubble(reduced_circ, labels =11.06)

?GOBubble

dev.off()

write.table(reduced_circ,file=paste0("1.","GO_circ.xls"),sep="\t",quote=F,col.names=T)

####热图

ROS=ROS[order(as.numeric(as.vector(ROS$logFC))),]

diffGene=as.vector(rownames(ROS))

afExp=rt[diffGene,]##需要重新调整下PI3K_symbolexpress

afcon=4

conData=data[,as.vector(colnames(data)[1:afcon])]

aftreat=afcon+1

treatData=data[,as.vector(colnames(data)[aftreat:ncol(data)])]

rt=cbind(conData,treatData)

conNum=ncol(conData)

treatNum=ncol(treatData)

Type=c(rep("con",conNum),rep("treat",treatNum))

names(Type)=colnames(rt)

Type=as.data.frame(Type)

anncolor=list(Type=c(con="#016DDB",treat="#E43401"))

pdf(file="ROS2_heatmap.pdf",height=7,width=13)

pheatmap(afExp, #热图数据

annotation=Type, #分组

color = colorRampPalette(c("#016DDB","white","#E43401"))(10), #热图颜色

cluster_cols =F, #不添加列聚类树

show_colnames =T, #展示列名

scale="row",

fontsize = 10,

fontsize_row=6,

fontsize_col=8,

annotation_colors=anncolor

)

dev.off()
